# Constructing chemical stable 4-carboxyl-quinoline linked covalent organic frameworks via Doebner reaction for nanofiltration

**DOI:** 10.1038/s41467-022-30319-2

**Published:** 2022-05-12

**Authors:** Yongliang Yang, Ling Yu, Tiancheng Chu, Hongyun Niu, Jun Wang, Yaqi Cai

**Affiliations:** 1 State Key Laboratory of Environmental Chemistry and Ecotoxicology, Research Center for Eco-Environmental Sciences, Chinese Academy of Sciences, 100085 Beijing, China; 2 University of Chinese Academy of Sciences, 100049 Beijing, China; 3 Institute of Oceanic and Environmental Chemical Engineering, Center for Membrane and Water Science &Technology, State Key Lab Breeding Base of Green Chemical Synthesis Technology, Zhejiang University of Technology, 310014 Hangzhou, China; 4 State Key Laboratory of Environmental Aquatic Chemistry, Research Center for Eco-Environmental Sciences, Chinese Academy of Sciences, 100085 Beijing, China; 5 National Engineering Research Center of Industrial Wastewater Detoxication and Resource Recovery, Research Center for Eco-Environmental Sciences, Chinese Academy of Sciences, 100085 Beijing, China; 6 School of Environment, Hangzhou Institute for Advanced Study, UCAS, Hangzhou, 310024 China

**Keywords:** Organic molecules in materials science, Polymers, Polymers

## Abstract

Covalent linkages are the key component of covalent organic frameworks (COFs). The development of stable and functional linkages is essential to expand the COFs family and broaden their application prospects. In this work, we report the synthesis of crystalline and chemical stable 4-carboxyl-quinoline linked COFs (QL-COFs) via Doebner reactions in both one-pot (OP) and post-synthetic modification (PSM) methods. Both methods can be universally applied to most of the reported imine COFs family via bottom-up construction or linkage conversion. Owing to the contractive pore size, more hydrophilic structure and better chemical stability than the conventional imine COFs endowed by 4-carboxyl-quinoline linkage, QL-COFs are supposed to possess a wider application range. We further demonstrate the nanofiltration membrane (NFM) based on QL-COF exhibited a desirable separation capacity with high rejection for small dye molecules (> 90%), high water permeance (850 L m^−2^ h^−1^ MPa^−1^) and tolerance of extreme conditions (1 M HCl/NaOH), which were benefitted from the enhanced properties of QL-COFs. Additionally, efficient ion sieving properties were also achieved by QL-COF membrane. We anticipate that this work opens up a way for the construction of robust and functional COFs materials for practical applications.

## Abstract (web summary)

Covalent linkages are the key component of covalent organic frameworks (COFs) and the development of stable and functional linkages is essential to broaden their application prospects. Here, the authors report the synthesis of crystalline and ultrastable 4-carboxyl-quinoline linked COFs via Doebner reactions in both one-pot and post-synthetic modification methods and demonstrate application of the COFs in nanofiltration.

## Introduction

Covalent organic frameworks (COFs) are crystalline porous organic polymer constructed by organic building blocks via covalent linkages^1–4^. So far, most of the studies in this field have focused on the design and regulation of organic building blocks^5^, however, the impact of linkage bonds on the properties and functions of COFs can not be neglected. In recent years, the exploration of robust linkages for COFs has become a research hotspot in the context of the unsatisfactory chemical stability and function of traditional reversible imine-linked COFs^6^. In addition to several stable linkages based on the irreversible condensation reactions, such as amide^7^, olefin^8,9^ and dioxin linkages^10,11^, it is worth noting that a type of imine-derived robust linkages have also been developed based on the chemical activity of the imine bond^12^, such as β-ketoenamine^13^, imidazole^14^, thiazole^15,16^, oxazole^17,18^ and quinoline^19,20^. The imine-derived linkages were usually synthesized by two-step reactions of reversible Schiff-base condensation and irreversible transformation of imine via OP and PSM methods. In this way, the chemical stability of COFs was greatly enhanced by the locking of the reversible imine bond under the premise of high crystallinity. A variety of fascinating functions can also be endowed by these linkages. Unfortunately, it’s worth noting that most of the reported synthesis of stable-linked COFs relied on the unique design of monomers, which increased the complexity of monomer synthesis and greatly limited the diversity of COFs. Therefore, it is promising to develop a synthesis scheme for stable linkage of COFs that is universal for the imine COFs family.

Nanofiltration is a liquid-phase pressure-driven membrane separation technology, which has been widely used for regeneration of water resource, including wastewater treatment and seawater desalination^21^. The conventional organic polymer nanofiltration membranes (NFMs) usually possess amorphous and dense structure with poor porosity, which limits the permeation of solvents. As a kind of crystalline porous materials, COFs possess the designable and ordered pore channels whose chemical structure can also be modified easily via PSM methods, which imply more permeation pathways and adjustable separation selectivity of COFs membranes^22^. Therefore, the application potential of COFs materials in the field of membrane separation is worth exploring. After the report by Banerjee’s group^23^, various COFs NFMs have been developed, which are mainly based on imine linkage^24–26^ and β-ketoenamine linkage^27–29^. However, the poor chemical stability of the reversible imine-linked COFs NFMs might hinder their practical application. The stable β-ketoenamine linked COFs were derived from 1,3,5-triformylphloroglucinol monomer, which greatly limited the design space in the structure and function of COFs NFMs. In this setting, developing robust linkages of COFs is a promising direction to broaden the diversity of COFs NFMs.

**Fig. 1:**
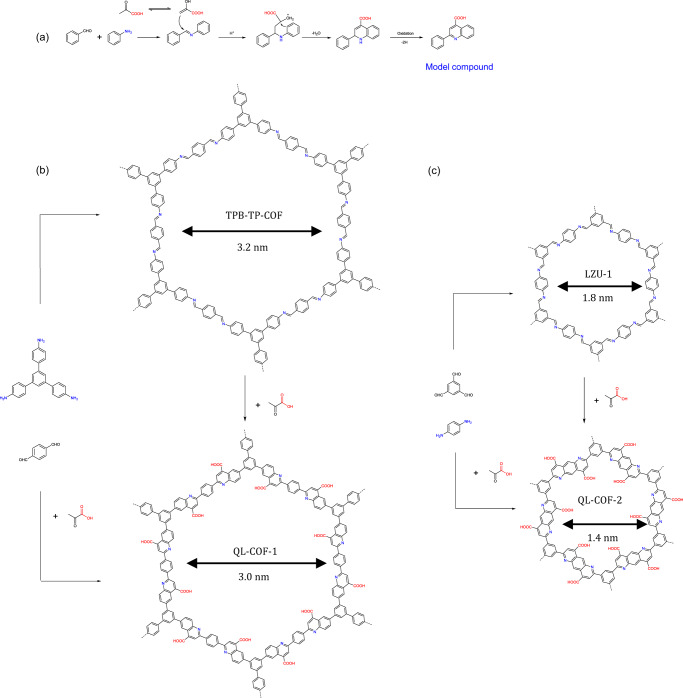
Synthesis of 4-carboxylquinoline linked QL-COF-1&2. **a** Proposed mechanism of the model compound formation via Doebner reaction. **b** and **c** Synthesis of QL-COF-1&2 in one-pot and post-synthetic modification.

Herein, we report the synthesis of 4-carboxyl-quinoline linked COFs (QL-COFs) via three-component Doebner reactions (Fig. 1). Owing to the reversible-irreversible sequence of the Doebner reaction mechanism, the aldehydes, amines monomers and pyruvic acid were assembled to give crystalline and porous framework products. QL-COFs can also be constructed by the linkage conversion from imine COFs. It is noted that the construction of QL-COFs possessed the potential to be extended to most of the imine family. Moreover, the 4-carboxyl-quinoline linkage endowed QL-COFs interesting characteristics including the contractive pore size, superior chemical stability and increased hydrophilicity. On this basis, QL-COF membranes were applied to the selective ion sieving and dye separation by nanofiltration, exhibiting remarkable size selectivity, high water permeance and durability in extreme conditions.

## Results and discussion

### Synthesis of QL-COF-1 in one-pot

The mechanism of the three-component Doebner quinoline synthesis reaction can be roughly divided into two steps: reversible formation of imine and irreversible formation of quinoline, which is suitable for the synthesis of crystalline and robust linked COFs. The quinoline-linked QL-COF-1 was firstly synthesized through the one-pot Doebner reaction between 1,3,5-tris(4-aminophenyl)benzene (TPB), terephthaldehyde (TP) and pyruvic acid in the solvent mixture of o-DCB/n-BuOH (1:1, v/v) at 120 °C for 72 h. The addition of glacial acetic acid was necessary for the successful synthesis of QL-COF-1 and the optimal aromatization oxidant was O_2_ (Supplementary Table 1).

### Characterization of QL-COF-1

**Fig. 2:**
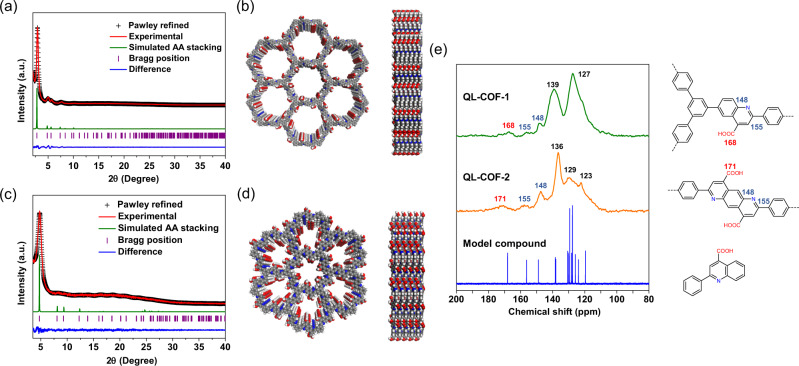
Crystal and chemical structure of QL-COF-1&2. **a**, **c** PXRD patterns of QL-COF-1 and QL-COF-2. Comparison of the experimental PXRD patterns (red) with the Pawley refined pattern (black), simulated pattern for AA stacking (green) and difference plot (blue). **b**, **d** Views of AA stacking model for QL-COF-1 and QL-COF-2. **e** The ^13^C NMR spectra of QL-COF-1 (green), QL-COF-2 (orange) and model compound (blue). All above date were measured on powder samples.

The crystalline structure of QL-COF-1 was firstly determined by powder X-ray diffraction (PXRD) measurement. As shown in Fig. 2a, the PXRD pattern exhibited an intense peak at 2.90° and other minor peaks at 5.05°, 5.69° and 7.83°, which were corresponding to the reflections from the planes (100), (110), (200) and (210), respectively. The structure models of QL-COF-1 were simulated by Material Studio (v. 7.0), the diffraction pattern was consistent with the eclipsed AA-stacking arrangement (Supplementary Fig. 1). After Pawley refinement of experimental PXRD pattern, a unit cell with parameters a = b = 37.21 Å, c = 3.77 Å, α = β = 90°, γ = 120° in space group P6 and good agreement factors (R_wp_ = 5.94%, R_p_ = 3.77%) were obtained.

The formation of the 4-carboxyl-quinoline linkage of QL-COF-1 was verified by solid-state ^13^C cross-polarization magic angle spinning (CP/MAS) NMR, Fourier transform infrared (FT-IR) spectroscopy and X-ray photoelectron spectroscopy (XPS). In addition to aromatic peaks at 129 and 137 ppm, the ^13^C NMR spectra of QL-COF-1 showed the peaks at 148 and 155 ppm assignable to quinolyl carbon and the peak at 168 ppm assignable to the carboxylic carbon which were confirmed by the ^13^C NMR spectra of model compound, indicating the successful formation of 4-carboxyl-quinoline linkage in QL-COF-1 (Fig. 2e). The FT-IR spectra of QL-COF-1 showed the characteristic peaks at 1704 and 1603 cm^−1^ corresponding to the C=O bonds of carboxyl and typical bands for the quinolyl species^30^, respectively (Supplementary Fig. 2). In the XPS spectra of QL-COF-1, the characteristic peaks of N 1 *s* was located at 400.1 eV corresponding to the quinolyl N, which was obviously different from the imine N at 398.8 eV in TPB-TP-COF (Supplementary Fig. 3).

Nitrogen adsorption experiment at 77 K was used to investigate the permanent porosity of QL-COF-1 (Supplementary Fig. 4). QL-COF-1 showed a typical type-ΙV isotherm, and the sharp N_2_ uptake in the low-pressure region (P/P_0_ < 0.05) indicated its microporosity. The Brunauer–Emmett–Teller (BET) surface area of QL-COF-1 was 1404 m^2^ g^−1^, with total pore volumes (P/P_0_ = 0.99) of 1.11 cm^3^ g^−1^. Pore size distribution of QL-COF-1 was derived from the nonlocal density functional theory model as 3.0 nm, which showed a 0.4 nm shrink of pore diameter in comparison with 3.4 nm pore size of TPB-TP-COF. QL-COF-1 showed a spherical morphology in the image of field-emission scanning electron microscopy, which was similar to the imine-linked TPB-TP-COF (Supplementary Fig. 5). The interplanar d-spacing of ca. 3.1 nm was confirmed by high-resolution transmission electron microscopy (HR-TEM) image as well as the FFT pattern, which was corresponding to the (100) lattice plane in PXRD result (2θ = 2.87°) according to the Bragg’s law (Supplementary Fig. 6). The above results once again confirmed the high crystallinity of QL-COF-1 synthesized in one-pot method.

### Synthesis of QL-COF-1 in post-synthetic modification method

Since the diverse preparation methods and broad application prospects of imine COFs, it is of great potential to develop PSM method for imine COFs. Moreover, it is easy to control the conversion rate via the PSM method by regulating the amount of reactants, which enables the accurate functional regulation of COFs materials. Considering the reversible-irreversible mechanism of Doebner reaction, the stepwise synthesis of QL-COF-1 through the PSM method can also be expected. We further carried out the synthesis of QL-COF-1 (named QL-COF-1-PSM) via the linkage conversion method from as-synthesized imine-linked TPB-TP-COF. The product obtained in the same reaction conditions as the OP method exhibited poor crystallinity (Supplementary Fig. 7). We speculate that the original crystalline framework structure of imine COFs would be partially destructed due to the structural distortion caused by the nucleophilic attack of pyruvic acid and the subsequent cyclization process, which led to the poor crystallinity.The presence of water in the reaction system could increase the reversibility of the imine linkages of COFs, which enables the rearrangement of amorphous polymer into crystalline structures^31^, therefore, we tried to substitute aqueous acetic acid for glacial acetic acid in the reaction system. As expected, the obtained PSM conversion product showed a high crystallinity. However, FT-IR spectrum indicated a poor conversion rate of imine linkage (Supplementary Fig. 7). Considering enol pyruvic acid is the active species for nucleophilic attack on imine in the Doebner reaction (Fig. 1a), the lower conversion of imine should be attributed to the weak acidity of aqueous acetic acid which is not conducive to the conversion of keto-enol form of pyruvic acid. Therefore, we further introduced p-toluenesulfonic acid (TsOH) into the reaction. On the one hand, the enhancement of the acidity of the reaction system is conducive to formation of enol pyruvate acid, on the other hand, the protonation rate of imine was increased, which were conducive to the nucleophilic addition of pyruvate acid to the imine carbon. In addition, oxygen was still the most suitable oxidation aromatization as same as OP method.

The high crystallinity of the imine TPB-TP-COF was mainly retained on the obtained QL-COF-1-PSM (Supplementary Fig. 7). QL-COF-1-PSM exhibited a similar ^13^C NMR spectrum with QL-COF-1 synthesized in one-pot (QL-COF-1-OP), the disappearance of the imine C signal at 158 ppm and the appearance of the carboxyl carbon signal at 168 ppm demonstrated the transformation of the linkage (Supplementary Fig. 8). Moreover, in the FT-IR spectra, the disappearance of imine C=N stretch at 1620 cm^−1^ and the appearance of new peaks at 1710 and 1603 cm^−1^ corresponding to the C=O bonds of carboxyl and typical peaks for the quinolyl species confirmed once again the transformation of imine linkage to 4-carboxyl-quinoline linkage (Supplementary Fig. 2). QL-COF-1-PSM exhibited porosity with high BET surface area (1455 m^2^ g^−1^) (Supplementary Fig. 4), which slightly lower than that of TPB-TP-COF (1720 m^2^ g^−1^) due to the contractive pore size. All above results demonstrated the feasibility of the PSM linkage conversion method.

### Synthesis of QL-COF-2

To investigate the scope for the synthesis of QL-COFs based on Doebner reactions, we performed the synthesis of QL-COF-2 in both OP and PSM methods. Both QL-COF-2-OP&PSM exhibited high crystallinity (Fig. 2), and the QL-COF-2-PSM show the retention of the high crystallinity of imine LZU-1 (Supplementary Fig. 9). As similar as QL-COF-1, the weakness of C=N stretch at 1620 cm^−1^ and presence of C=O bonds of carboxyl and quinolyl species vibration band at 1704 and 1602 cm^−1^ were founded in FT-IR spectra of QL-COF-2 (Supplementary Fig. 10). In addition,^13^C NMR spectra of QL-COF-2 showed the disappearance of the imine C signal and the appearance of the carboxyl carbon signal (Supplementary Fig. 11). Both of above results demonstrated the formation of the 4-carboxylquinoline linkage. XPS N 1 *s* spectra indicated that the proportion of quinoline linkage in QL-COF-2-OP and -PSM were 90.2% and 87.0%, respectively (Supplementary Fig. 12).

Nitrogen adsorption experiment indicated that QL-COF-2 possessed good porosity, the BET surface areas of QL-COF-2-OP and -PSM were 390 and 399 m^2^ g^−1^ (Supplementary Fig. 13). As similar as QL-COF-1, QL-COF-2 showed a significantly contractive pore diameter of 1.3 nm compared to imine LZU-1 (1.8 nm) due to the conversion of imine linkage. The decreased BET surface area from imine LZU-1 (459 m^2^ g^−1^) to QL-COF-2-PSM after linkage conversion can also be attributed to the reduction in pore size. The HR-TEM images of QL-COF-2 (Supplementary Fig. 14) exhibited the spherical morphology. The ordered lattice fringe can be well distinguished, the interplanar d-spacing was measured as 1.79 nm which was consistent with the FFT pattern. Moreover, the PXRD diffraction peaks corresponding to the (100) lattice plane in QL-COF-2 (2θ = 4.88°) were also coincident with the d-spacing result according to the Bragg’s law, manifesting the high crystallinity of QL-COF-2.

### Stability and hydrophilicity of QL-COFs

**Fig. 3:**
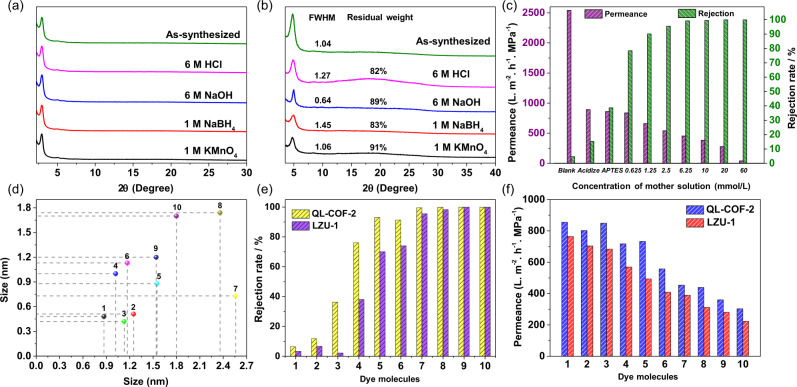
Stability test of QL-COFs and nanofiltration performance test of QL-COF-2 membrane. PXRD patterns measured after one day treatment of QL-COF-1 (**a**) and QL-COF-2 (**b**) in 6 M HCl (pink), 6 M NaOH (blue), 1 M NaBH_4_ (red) and 1 M KMnO_4_ (black). **c** The water permeances and dye rejection rates of blank tube and the QL-COF-2 membranes synthesized at different concentration of the mother solution in the nanofiltration experiments of 50 ppm Congo red. **d** Serial numbers and molecular sizes of dyes in nanofiltration experiments. 1-Neutral red, 2-methylene blue, 3-methyl orange, 4-acid blue, 5-chrome black T, 6-acid fuchsin, 7-Congo red, 8-methyl blue, 9-rose bengal, 10-vitamin B-12. **e** Rejection rates of various dyes through the QL-COF-2 and LZU-1 membranes. **f** Water permeances of various dyes through the QL-COF-2 and LZU-1 membranes.

In order to test the chemical stability, QL-COFs and corresponding imine COFs were subjected to various harsh conditions, including strong acid (6 M HCl), strong base (6 M NaOH), strong oxidant (1 M KMnO_4_) and strong reducing agent (1 M NaBH_4_). Both imine-linked TPB-TP-COF and LZU-1-COF showed obvious loss of crystallinity under such extreme conditions within 24 h, and LZU-1 even decomposed very quickly (within 30 min) under a weaker acidic condition (0.1 M HCl) (Supplementary Fig. 15). In contrast, QL-COF-1&2 showed superior tolerability to all the extreme conditions due to the good chemical stability of the quinoline linkage. After 24 h treatment under the above conditions, both QL-COF-1&2 showed the retention of crystallinity, chemical structure and porosity according to the PXRD (Fig. 3a, b), FT-IR and BET results (Supplementary Figs. 16 and 17). It is noted that there are indeed a broadening and a slight decrease in intensity of the (100) peaks of QL-COF-2 in Fig. 3b, which is due to the existence of small amount of imine linkages in QL-COF-2 demonstrated by the XPS results (Supplementary Fig. 12). According to the stability test of imine-linked LZU-1 (Supplementary Fig. 15b), it can be seen that the imine-linked LZU-1 can generally withstand 6 M NaOH but cannot withstand the other three conditions, which explains the narrower (100) peak width of QL-COF-2 after treated with NaOH. Thermogravimetric analysis indicated that QL-COF-1/-2 were thermally stable up to 300 °C, respectively, (Supplementary Fig. 18) which were weaker than the imine TPB-TP-COF and LZU-1 (up to 400 °C). Considering that the critical thermal stability temperature of 4-phenylquinoline linked COFs synthesized by Povaorv reactions^19^ was also about 300 °C, the weaker thermal stability maybe result from the intrinsic chemical structure of the quinoline linkage. In addition, it has been reported that the decarboxylation of quinolinecarboxylic acid could be occurred at 280–290 °C^32,33^, which would also lead to the weight loss. In fact, we believe that the slightly weaker thermal stability does not significantly limit the application prospects of QL-COFs. The water contact angle of QL-COF-2 (64.45°) was smaller than LZU-1 (77.27°), which suggested the higher hydrophilicity of QL-COF-2, implying the better permeability of water molecules (Supplementary Fig. 19).

### Synthesis and characterization of QL-COF-2 membranes

Owing to the superior chemical stability, the constricted aperture size and the good hydrophilicity endowed by 4-carboxyl-quinoline linkage, the QL-COF-2 membrane was supposed to exhibited superior performance in nanofiltration. Commercial alumina ceramic membrane tubes were chosen as the substrates due to their good stability and low cost. The Al_2_O_3_ membrane tubes were firstly modified with 3-aminopropyltriethoxysilane (APTES), and then reacted in the mother solution (SI, Section 2.2) for QL-COF-2 synthesis at 120 °C for 3 days to obtain the QL-COF-2 active layer on the surface of Al_2_O_3_ membrane tubes. The white Al_2_O_3_ membrane tubes all turned brown after the reaction (Supplementary Fig. 20), which preliminarily demonstrated that the QL-COF-2 were modified on the surface of the substrate in situ. In addition, the higher concentration of the mother solution, the darker of the QL-COF-2 membrane. The SEM images (Supplementary Fig. 22) further proved the successful fabrication of QL-COF-2 membranes. It was obvious that the COFs were grown on the surface of the substrate evenly and densely without any obvious defects and pinholes when the mother solution concentration was higher than 6.25 mmol/L (calculated as functional group). The SEM images of the cross-section of the membrane tubes indicated that the thickness of the QL-COF-2 membranes were positively correlated with the concentration of the reaction mother solution (Supplementary Fig. 23). The FT-IR spectra of QL-COF-2 membranes with different thickness matched well with that of QL-COF-2-OP, confirming the successful fabrication of QL-COF-2 membranes on Al_2_O_3_ substance (Supplementary Fig. 24). In addition, according to Dichtel’s report^25^, the crystallinity can only be confirmed in a thick film (>100 μm). Due to the only several microns thickness of QL-COF-2 membranes, the XRD pattern showed the weak diffraction peaks corresponding to QL-COF-2 membranes (Supplementary Fig. 25). The PXRD pattern of the sediment in the preparation reactor of QL-COF-2 membrane showed the high intensity of diffraction peak and the same diffraction peak position with QL-COF-2 membrane, which implied the high crystallinity of the synthesized QL-COF-2 membrane.

### Nanofiltration performance of QL-COF-2 membranes

The nanofiltration performance of the QL-COF-2 membranes were evaluated using a cross-flow filtration circulation system at 25 °C under a transmembrane pressure of 5 bar (Supplementary Fig. 26). Firstly, the separation performance of the QL-COF-2 membranes in the 50 ppm Congo red (CR) (Mw = 696.68; ca. 2.56 nm × 0.73 nm) were investigated. As shown in Fig. 3c, the pristine Al_2_O_3_ membrane tube showed a water permeance around 2540 L m^−2^ h^−1^ MPa^−1^ with a negligible CR rejection (4.6%). The water permeance of the pristine Al_2_O_3_ membrane tube significantly decreased to 892 L m^−2^ h^−1^ MPa^−1^ after acidification. The SEM image of the membrane surface (Supplementary Fig. 21) showed that the surface porosities of the Al_2_O_3_ membrane tubes were significantly reduced after acidification. We speculate that the porous γ-alumina layer was slightly dissolved in acid (1 M HCl), and the pores of the original porous alumina layer were plugged by the aluminum hydroxide generated during the water washing and drying, which lead to the significant decrease of water permeance. After the modification of APTES, the dye retention rate only slightly increased to 38.6%. However, when the QL-COF-2 membranes were grafted onto the substrate, the rejection rates of the dye were significantly enhanced to more than 78.4%, indicating the crucial role of COF active layer in molecular sieving. As the concentration of mother solution increased to 6.25 mmol/L, the defects of COFs membrane were reduced and the rejection rate of CR was enhanced to 99.9%, which were consistent with the SEM results. However, a further increase in the concentration of the mother solution cannot significantly enhance the CR rejection rate but would lead to the augment in the thickness of COFs layer, resulting in the increase of the mass transfer resistance of water molecules and hence led to the decrease of water permeability. To balance effective rejection of dye (>99%) and high water permeance, the appropriate concentration of the reaction mother solution was set at 6.25 mmol/L.

Recently, the controversy over the mechanism of dyes retention by COFs membrane has received widespread attention. Dichtel et al. demonstrated that adsorption is the primary mechanism driving rejection and separation of dyes in thick, polycrystalline COF membranes^34^. It is worth noting that there are several typical characteristics of the adsorption membrane: (i) high quality (100 mg) and thickness (several hundred microns), exhibiting high adsorption capacity to remove dyes; (ii) the rejection rate of dyes decreased as the total passed volume increased; (iii) high rejection rate of dyes were only achieved at a low flow rate (0.5 mL/min), due to the slow adsorption process of the membrane to the dyes; (iv) the separation of the dyes from the membrane depended on the difference in adsorption affinity. By contrast, in this work, it is the thin film with ~5 μm thickness and <1 mg mass for QL-COF-2 membrane when the concentration of mother solution is 6.25 mmol/L. The adsorption capacity of QL-COF-2 to CR in the 50 mg L^−1^ solution was measured as only 40.88 mg g^−1^ (Supplementary Fig. 27). In the nanofiltration performance test, the CR molecule in ~140 mL of 50 mg L^−1^ feed solution can be effectively rejected in 20 min (>99.9%) at a high flow rate of ~7 mL/min, which is far greater than the adsorption capacity of QL-COF-2 membrane. Hereby, it is impossible for QL-COF-2 to act as an adsorbent in the nanofiltration experiments.

The separation property of the QL-COF-2 membrane was further investigated by using several dye molecules with different sizes, including vitamin B-12, rose bengal, methyl blue, CR, acid fuchsin, chrome black T, acid blue, methylene blue, methyl orange and neutral red (Fig. 3d). Imine-linked LZU-1 membrane was also synthesized under the same condition as a comparison. The rejection rates of the dyes showed a significant correlation with the molecular weight of the dyes but no correlation with the adsorption affinity to QL-COF-2 (Supplementary Fig. 29). Meanwhile, as shown in Fig. 3e, dye molecules with the sizes larger than 1.3 nm were generally rejected (>90%) by QL-COF-2 membrane. Considering the 1.3 nm aperture size of QL-COF-2, we confirmed that the size exclusion effect was dominated in the rejection of dyes. By contrast, the rejection of all these dyes on QL-COF-1 membrane were pretty low (<60%), which should be attributed to the large pore size (3.0 nm) (Supplementary Fig. S30). It is worth noting that the electrostatic effect also played an important role in dye rejection. The QL-COF-2 membrane exhibited a higher rejection for negatively charged MO than that of the positively charged MB although the molecular size of MO (1.13 × 0.42 nm) is smaller than MB (1.25 × 0.51 nm). It was mainly benefitted from the negatively charged membrane surface (−58.9 mV) endowed by the carboxyl groups of the QL-COF-2 (Supplementary Fig. 31). In addition, the QL-COF-2 membrane also exhibited a higher rejection for all dyes than LZU-1 membrane with a superior water permeability (Fig. 3f), which can be attributed to the contractive pore size and better hydrophilicity provided by 4-carboxyl-quinoline linkage.

**Fig. 4:**
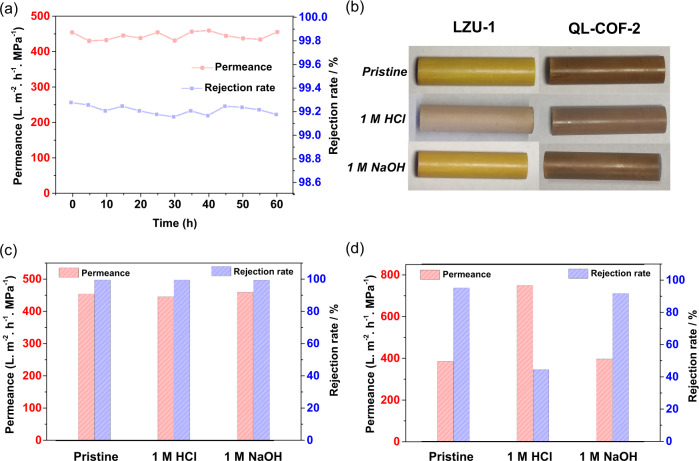
Stability and durability of the QL-COF-2 and LZU-1 membranes. **a** Long-term stability of QL-COF-2 membranes under 50 ppm CR solutions. **b** The images of QL-COF-2 and LZU-1 membranes before and after treatment in 1 M HCl and 1 M NaOH. Nanofiltration performance of QL-COF-2 (**c**) and LZU-1 (**d**) membranes under harsh treatment.

QL-COF-2 membrane exhibited stable and continuous dye rejection with almost constant flux during 60 h at 5 bar (Fig. 4a), even after the treatment in 1 M HCl or 1 M NaOH for 24 h (Fig. 4c). It implied the high durability and wide application of QL-COF-2 membrane. However, the decomposition of LZU-1 membrane was observed only after a 30 min treatment of 1 M HCl with significantly reduced rejection of CR and increased water flux (Fig. 4d), which further indicated the significance of enhanced chemical stability of the QL-COF-2 membrane achieved by construction of the robust linkages.

### Cation sieving performance of QL-COF-2 membrane

COFs membranes exhibited great potential in the sieving of organic molecules^23,35^ and ions^27,36^, benefited from their definite and ordered 1D nanochannels. Given the intrinsic 1.3 nm channel with negative charge introduced by carboxylate groups, we have developed the cation selective sieving function of QL-COF-2 membrane. QL-COF-2 was ultrasonically dispersed in alkaline water, and then filtered on a porous anodic aluminum oxide (AAO) membrane under reduced pressure to fabricate the QL-COF-2 membranes (Supplementary Fig. 32). The cross-sectional SEM images (Supplementary Fig. 33) showed that the thickness of the film was positively correlated with the amount of COF, and the surface SEM images (Supplementary Fig. 34) indicated that the dense and defect-free COF film with 45 mm diameter required above 10 mg COF. Cation selective sieving experiments were carried out using various tetraalkyl ammonium (Me_4_N^+^, Et_4_N^+^, Bu_4_N^+^, Hex_4_N^+^, Oct_4_N^+^, Dodec_4_N^+^) with different sizes^36^. In order to control the interference of anions, the high molecular weight polystyrenesulfonate (~ 70 kDa) was selected as the anion. The same volume of tetraalkyl ammonium polystyrenesulfonate salts methanol solution was separated by the membranes in the H-shaped electrolytic cell. The conductivity measured between the two sides of the membranes were used to define the transmembrane resistance of ions, so that the ion sieving performance of the membrane was evaluated accordingly. (See the SI, Section S6).

**Fig. 5:**
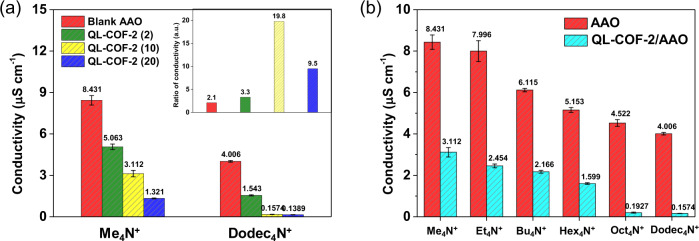
Cation sieving experiments. **a** Transmembrane conductivity of Me_4_N^+^ and Dodec_4_N^+^ performed by blank AAO membrane and QL-COF-2/AAO membrane with different mass of COF. Inset: the conductivity ratio of Me_4_N^+^ to Dodec_4_N^+^ across different membranes. **b** Transmembrane conductivity of six cations across blank AAO and QL-COF-2 (10) membranes. Error bars donate standard deviation.

Firstly, we have investigated the conductivity of tetramethylammonium (Me_4_N^+^, diameter = 2.36 Å) and tetradodecylammonium (Dodec_4_N^+^, diameter = 24.7 Å) across the blank AAO membrane and QL-COF-2 membranes with different thicknesses. As shown in Fig. 5a, the conductivity of Me_4_N^+^ is 2.1 times that of Dodec_4_N^+^ in the control experiment carried out by the AAO membrane, which was originated from the intrinsic difference in molar conductivity of the two cations^37^. The conductivity of both Me_4_N^+^ and Dodec_4_N^+^ decreased with the enhanced thickness of QL-COF-2 membrane due to the increased mass transfer resistance to across the membrane. The maximum conductivity ratio of Me_4_N^+^ and Dodec_4_N^+^ was achieved when the COF membrane was 10 mg [QL-COF-2 (10)], implying the best ion sieving performance. A further increase of the membrane thickness only enhanced the mass transfer resistance of Me_4_N^+^ without significantly reducing the transmembrane conduction of Dodec_4_N^+^. Subsequently, the transmembrane conductivity of six alkylammonium cations with different sizes were measured to evaluate the size sieving function of the QL-COF-2 (10) membrane (Fig. 5b). The conductivity reduction factors (ratio of conductivity across AAO and QL-COF-2 membranes) of the small cations (Me_4_N^+^, Et_4_N^+^, Bu_4_N^+^, Hex_4_N^+^) were about 3, while the factors of large cations (Oct_4_N^+^, Dodec_4_N^+^) were above 20. Furthermore, the transmembrane conductivity of large cations was below 0.2 μS cm^−1^, implying that the cations were almost rejected by the QL-COF-2 membrane. Given that the pore size of COFs (diameter = 14 Å) is larger than the size of small ions (Hex_4_N^+^ diameter = 13.4 Å) but smaller than that of large ions (Oct_4_N^+^ diameter = 17.8 Å), the difference in the transmembrane conductivity of cations was mainly resulted from the size exclusion effect. All above results indicated that QL-COF-2 membrane possess size-selective sieving function for ions.

In conclusion, we have developed crystalline, porous and chemical stable 4-carboxyl-QL-COFs based on the three-component Doebner reaction. The precise covalent assembly of the 4-carboxyl-quinoline linkage were realized in QL-COF-1&2 in both OP and PSM methods, implying the expansion potential of this linkage in the imine-COFs family. Due to the high chemical stability, shrinking pore size and improved hydrophilicity endowed by this linkage, QL-COF-2 membrane showed higher flux and persistent molecular rejection in the nanofiltration of the dye solutions as compared to imine LZU-1 membrane. In addition, efficient size-selective cation sieving can also be achieved by QL-COF-2 membrane with negatively charged nanochannel. We anticipate that this kind of 4-carboxyl-quinoline-linked COFs with assembly diversity and carboxyl-based post-modification potential possessed broad application prospects in nanofiltration and other fields.

## Methods

### Synthesis of QL-COF-1 in one-pot

A 25 mL Schlenk tube was charged p-phthalaldehyde (48.3 mg, 0.36 mmol), 1,3,5-TPB (84 mg, 0.24 mmol), o-dichlorobenzene (1.5 mL), n-butyl alcohol (1.5 mL), glacial acetic acid (100 µL). The tube was firstly sonicated for 30 min, then pyruvic acid (50 µL, 0.72 mmol) was added with vortex. Subsequently, the tube charged with oxygen with three degassing-filling cycle using Schlenk line under 77 K in liquid N_2_ bath and sealed. The reaction was heated at 120 °C for 3 days yielding precipitate which was isolated by suction filtration, washed with methanol (30 ml × 3), ethyl acetate (30 ml × 3) and n-hexane (15 ml × 3) and drying under nitrogen flow at room temperature for 2 h.

### Synthesis of QL-COF-1 in post-synthetic modification

A 25 mL Schlenk tube was charged TPB-TP-COF (56 mg), o-dichlorobenzene (1 mL), n-butyl alcohol (1 mL), 6 M acetic acid aqueous solution (200 µL), TsOH (20 mg) and pyruvic acid (28 µL, 0.4 mmol). The tube was sonicated for 30 min, then charged with oxygen with three degassing-filling cycle using Schlenk line under 77 K in liquid N_2_ bath and sealed. The reaction was heated at 120 °C for 3 days yielding precipitate which was isolated by suction filtration, washed with methanol (30 ml × 3), ethyl acetate (30 ml × 3) and n-hexane (15 ml × 3) and drying under nitrogen flow at room temperature for 2 h.

### Synthesis of QL-COF-2 in one-pot

A 25 mL Schlenk tube was charged 1,3,5-triformylbenzene (48 mg, 0.30 mmol), 1,4-diaminobenzene (48 mg, 0.45 mmol), glacial acetic acid (100 µL) and 1,4-dioxane (3 mL). After sonicating for 30 min, pyruvic acid (62.5 µL, 0.9 mmol) was added with vortex. Subsequently, the tube charged with oxygen with three degassing-filling cycle using Schlenk line under 77 K in liquid N_2_ bath and sealed. The reaction was heated at 120 °C for 3 days yielding precipitate which was isolated by suction filtration, washed with acetone (30 ml × 3) and THF (30 ml × 3) and evacuated under vacuum at 120 °C for 10 h.

### Synthesis of QL-COF-2 in post-synthetic modification

A 25 mL Schlenk tube was charged LZU-1 (96 mg), 1,4-dioxane (3 mL), 6 M acetic acid aqueous solution (300 µL), TsOH (20 mg) and pyruvic acid (69.5 µL, 1 mmol). The tube was sonicated for 30 min, then charged with oxygen with three degassing-filling cycle using Schlenk line under 77 K in liquid N_2_ bath and sealed. The reaction was heated at 120 °C for 3 days yielding precipitate which was isolated by suction filtration, washed with acetone (30 ml × 3) and THF (30 ml × 3) and evacuated under vacuum at 120 °C for 10 h.

### Synthesis of the Al_2_O_3_ tube supported QL-COF-2 and LZU-1 membrane

The Al_2_O_3_ ceramic membrane tube was immersed in 1 M HCl solution overnight to activate the surface hydroxyl groups. After neutralized and dried, the tube was reacted with APTES (0.6 mM in 30 mL of toluene) at 110 °C for 2 h to obtain amino-modified tube. The QL-COF-2 membrane was prepared by in situ growth through solvothermal reaction in the series of mother solution (see the Supplementary Table 2). After 72 h reaction at 120 °C under oxygen atmosphere, the membrane tube was washed with acetone (30 ml × 3), THF (30 ml × 3) and water (30 ml × 3) under ultrasonic.

The LZU-1 membrane was synthesized in the same procedure without addition of pyruvic acid.

### Dyes rejections by nanofiltration

The rejection performances of COFs membrane were measured by nanofiltration process using a cross-flow filtration device. The membrane tube was equipped into a home-made tubular module, and then a dye aqueous solution (50 mg/L) was recycled by a diaphragm pump with operating pressure of 5 bar. Before measurement, the filtration system was cycled 0.5 h to reach a steady state. The water permeance was determined by the volume of permeate collected during 20 min. The concentrations of dyes in the feed and permeate were analyzed by a UV–vis detector to calculate the rejection rate.

## Supplementary information

Supplementary Information

## Data Availability

The data generated in this work are provided in the Source Data file and its Supplementary Information. Source data are provided with this paper.

## Source data

Source Data
