# Supplementary material for: Constructing chemical stable 4-carboxyl-quinoline linked covalent organic frameworks via Doebner reaction for nanofiltration
Source: Nat Commun. 2022 May 12;13:2615. doi: 10.1038/s41467-022-30319-2 (PMC9098490; doi:10.1038/s41467-022-30319-2)
Supplement: Supplementary file 1 — Supplementary Information [file 41467_2022_30319_MOESM1_ESM.pdf]

Supplementary Information

Constructing Chemical Stable 4-Carboxyl-quinoline Linked Covalent Organic Frameworks via Doebner Reaction for Nanofiltration

Yongliang Yang[1,2] Ling Yu[3] Tiancheng Chu[2,4,5], Hongyun Niu[1], Jun Wang[2,4,5]*, Yaqi Cai[1,2,6]*

[1] State Key Laboratory of Environmental Chemistry and Ecotoxicology, Research Center for Eco-Environmental Sciences, Chinese Academy of Sciences, Beijing, 100085, China

[2] University of Chinese Academy of Sciences, Beijing, 100049, China

[3] Institute of Oceanic and Environmental Chemical Engineering, Center for Membrane and Water Science &Technology, State Key Lab Breeding Base of Green Chemical Synthesis Technology, Zhejiang University of Technology, Hangzhou, 310014, China [4] State Key Laboratory of Environmental Aquatic Chemistry, Research Center for Eco-Environmental Sciences, Chinese Academy of Sciences, Beijing, 100085, China

[5] National Engineering Research Center of Industrial Wastewater Detoxication and Resource Recovery, Research Center for Eco-Environmental Sciences, Chinese Academy of Sciences, Beijing 100085, China

[6] School of Environment, Hangzhou Institute for Advanced Study, UCAS, Hangzhou 310024, China

* Corresponding author: E-mail: caiyaqi@rcees.ac.cn; junwang@rcees.ac.cn

Section 1 General Information

1.1 Chemicals and reagents

All reagents and solvents were purchased from commercial sources and used without further purification. Aminopropyltriethoxysilane (APTES), 1,4-diaminobenzene, p-phthalaldehyde, 1,4-dioxane, n-butyl alcohol and o-dichlorobenzene were purchased from J&K Chemical Co. Ltd. Glacial acetic acid, acetone, methanol, dichloromethane, n-hexane were obtained from Sinopharm Chemistry Reagent Co. Ltd. Pyruvic acid was purchased from TCI. 1,3,5-Triformylbenzene, 1,3,5-tris(4-aminophenyl)benzene were purchased from Jilin Chinese Academy of Sciences-Yanshen Technology Co., Ltd. Al_2O_3 ceramic membrane tubes (1 m-long, 8 mm inner diameter, 12 mm outer diameter) with 5 μm-thick γ-alumina layer (average pore size 0.1 μm) were purchased from JieXi LiShun Technology Co., China, then these tubes were cut into 5 cm-long. TPB-TP-COF and LZU-1-COF were synthesized according to the literature.[1, 2]

1.2 Characterization

Powder X-ray diffraction (PXRD, Bruker D8 ADVANCE, Germany) using a Cu Kα (λ = 1.5418 Å) radiation ranging from 5° to 40° with a resolution of 0.02° was utilized to analysis the crystalline. The microstructures of the catalysts were investigated by field emission scanning electron microscope (FE-SEM, SU8020, HITACHI) and high-resolution transmission electron microscope (HR-TEM, JEM-2100F, JEOL) at accelerating voltage of 200 kV. Surface analysis by X-ray photoelectron spectroscopy (XPS) was carried out using Thermo Fisher ESCALAB 250Xi equipment (Waltham, MA), and the X-ray source was Al Kα radiation (1486.6 eV, monochromatic). Fourier transform-infrared (FT-IR) spectra in the 4000–400 cm^{-1} region were recorded on a NEXUS 670 Infrared Fourier Transform Spectrometer (Nicolet Thermo, Waltham, MA). Solid-state nuclear magnetic resonance (ssNMR) spectroscopy was obtained on a JNM-ECZ600R spectrometer (JEOL). Surface area and pore volume were measured by BrunauerEmmett-Teller (BET) methods (ASAP2000 V3.01A; Micrometritics,

Norcross, GA). The rejection rate of dyes solution were measured by ultraviolet-visible spectrophotometer (UV-8000, METASHI, Shanghai, China). Zeta potentials were measured by nano-particle potentiometer (Zetasizer Nano, England). The hydrophilicity were measured by a contact angle meter (OCA50, Datephysics, Germany) through sessile drop method. Nanofiltration experiments were carried out by home-made cross-flow filtration system.

Section 2 Synthetic procedure

2.1 Synthesis of 2-phenylquinoline-4-carboxylic acid (model compound)[3]

The mixture of aniline (1.86 g, 20 mmol) and benzaldehyde (2.12 g, 20 mmol) in ethanol (30 mL) was refluxed for 1 h; pyruvic acid (2.6 g, 30 mmol) and trifluoroacetic acid (3 mL) were then added to the reaction mass and further refluxed for 72 h. Reaction completion was monitored by using TLC. The reaction mixture was poured into ice water (60 mL), the solid product was filtered and was added to the aqueous K_2CO_3. The solution was adjusted to basic, then filtered. The filtrate was acidified with diluted HCl to pH 1–2, and mixture was extracted with three 40 mL portions of CH_2Cl_2. The combined organic phase was washed with 40 mL water, dried over anhydrous sodium sulfate and concentrated under reduced pressure. The crude product was purified by using flash column chromatography.

^{1}H-NMR (400 MHz, DMSO-d6): δ 14.09 (s, 1H), 8.72 (d, J = 8.5 Hz, 1H), 8.19 (s, 1H), 8.09 (d, J = 7.9 Hz, 1H), 8.03 (d, J = 8.2 Hz, 1H), 7.96 (d, J = 7.3 Hz, 1H), 7.88 (t, J = 7.5 Hz, 2H), 7.78 (t, J = 7.6 Hz, 2H)

Supplementary Table 1. Optimization of synthesis conditions for QL-COF-1

**Acetic acid**	**Oxidant**	**Crystalline**
+	-	No
+	DDQ	No
+	DMSO	No
+	Chloranil	No
+	O_2	High
-	O_2	Low

Supplementary Table 2. The mother solutions for the synthesis of QL-COF-2 and LZU-1 membrane.

**Solvent**	**1,3,5-Triformylbenzene (mg)**	**1,4-Diaminobenzene (mg)**	**Pyruvic acid (μL)**	**c (mmol/L) (calculated as functional group)**
15 mL 1,4-dioxane + 1.5 mL 3 M HOAc	48	48	62.5	60
	16	16	20.8	20
	8	8	10.4	10
	5	5	6.5	6.25
	2	2	2.6	2.5
	1	1	1.3	1.25
	0.5	0.5	0.65	0.625

2.2 Synthesis of the Al_2O_3 tube supported QL-COF-1 membrane

The QL-COF-1 membrane was prepared by *in situ* growth through solvothermal reaction. A 25 mL Schlenk tube was charged p-phthalaldehyde (6.2 mg, 0.045 mmol), 1,3,5-tris(4-aminophenyl)benzene (10.85 mg, 0.03 mmol), o-dichlorobenzene (7.5 mL), n-butyl alcohol (7.5 mL), 3 M HAc (1.5 mL). The tube was firstly sonicated for 30 min, then pyruvic acid (6.5 μL, 0.09 mmol) was added with vortex. Then put the APTES modified Al_2O_3 membrane tube into the reaction tube. Subsequently, the tube charged with oxygen with three degassing-filling cycle using Schlenk line and sealed. The reaction was heated at 120 ºC for 3 days, washed with methanol (30 ml×3), DCM (30 ml×3) and n-hexane (15 ml×3) and drying under nitrogen flow at room temperature for 2 h.

Section 3 Structure Modeling and PXRD Refinement of QL-COF-1&2

Structural modeling of QL-COFs were generated using the Accelrys Materials Studio 7.0 software package. The space groups were obtained from the Reticular Chemistry Structure Resource. The model was constructed in the initial lattice with the space group

of P1. The proposed model was geometry optimized using the MS Forcite molecular dynamics module (Universal force fields, Ewald summations) to obtain the optimized lattice parameters. P6 and P3 were suitable for AA stacking model of QL-COF-1 and QL-COF-2, respectively. AB stacking of QL-COFs were examined by offsetting the stacked units from the AA model. Pawley refinement was applied to define the lattice parameters by Reflex module, producing the refined PXRD profiles. The simulated PXRD patterns of AA stacking are the best agreement with the experimental patterns of both QL-COF-1&2.

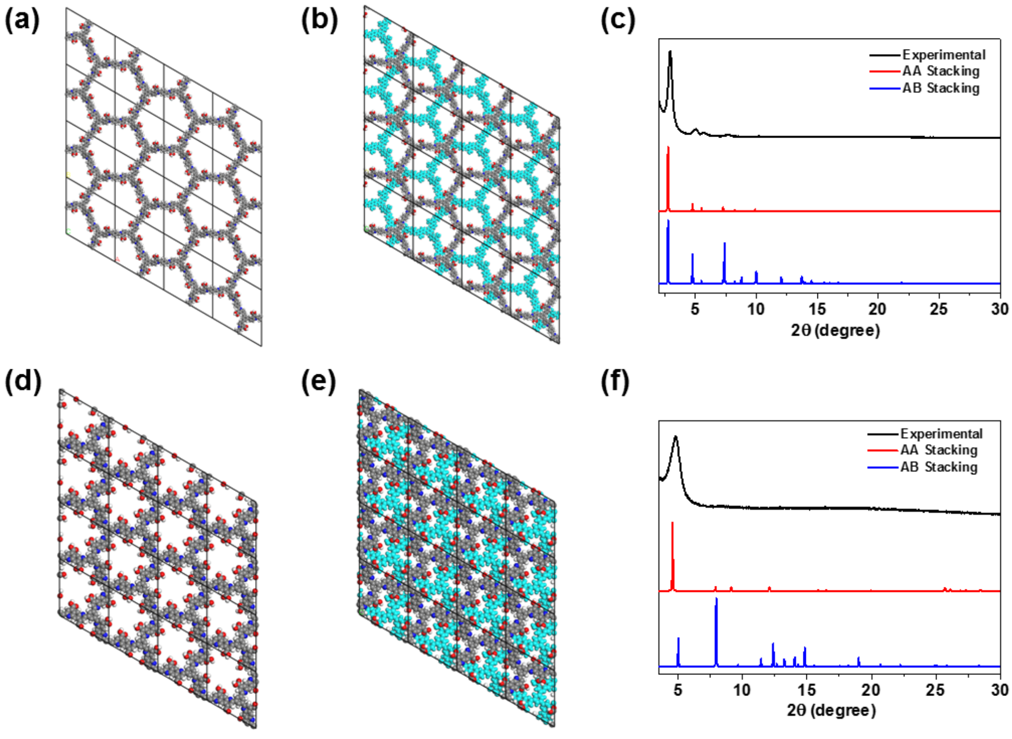

Supplementary Figure 1. The simulated model of QL-COF-1&2 as well as the comparison of PXRD patterns. The view of the simulated eclipsed AA-stacking (a), staggered AB-stacking (b) model of QL-COF-1 and eclipsed AA-stacking (d), staggered AB-stacking (e) model of QL-COF-2. Simulated PXRD patterns of AA (red) and AB (blue) stacking model compared to experimental pattern (black) of QL-COF-1 (c) and QL-COF-2 (f).

Supplementary Table 3. Atomistic coordinates for the Pawley-refined QL-COF-1.

QL-COF-1	Hexagonal, P6<br>a = b = 37.21 Å, c = 3.77 Å<br>α = β = 90°, γ = 120°		
Atom	x	y	z
C1	0.39185	0.82447	0.00637
C2	0.36893	0.78066	0.01042
C3	0.38373	0.75677	-0.15777
C4	0.42323	0.7774	-0.31622
C5	0.44734	0.82054	-0.30311
C6	0.43203	0.84404	-0.14498
C7	0.35788	0.71048	-0.16561
C8	0.37655	0.6857	-0.16729
N9	0.42967	0.54376	-0.13879
C10	0.4435	0.91104	0.00648
C11	0.48454	0.52788	0.0044
C12	0.52753	0.54279	-0.00105
C13	0.54272	0.5152	-0.00031
C14	0.47276	0.62284	0.15539
C15	0.48937	0.59617	0.15688
C16	0.49913	0.66531	0.30556
O17	0.49365	0.69398	0.22798
O18	0.53157	0.67302	0.53295
H19	0.34042	0.76457	0.15758
H20	0.43544	0.76046	-0.45711
H21	0.47781	0.83607	-0.42412
H22	0.40998	0.70044	-0.16797
H23	0.54957	0.57571	-0.01212
H24	0.57596	0.52749	-0.002
H25	0.51927	0.60647	0.27893
H26	0.54582	0.70293	0.61567

Supplementary Table 4. Atomistic coordinates for the Pawley-refined QL-COF-2.

**QL-COF-2**	**Hexagonal, P3**<br>**a = b = 22.01 Å, c = 3.68 Å**<br>**α = β = 90°, γ = 120°**		
Atom	x	y	z
C1	1.51887	1.22954	0.32187
N2	1.47564	1.25037	0.17872
C3	1.40608	1.20682	0.13407
C4	1.37476	1.13537	0.23576
C5	1.30148	1.09354	0.19585
C6	1.49061	1.15944	0.429
C7	1.41867	1.11059	0.38171
C8	1.27734	1.29095	-0.32948
O9	1.33432	1.32377	-0.47712
O10	1.23481	1.32114	-0.30161
C11	1.59472	1.28214	0.3546
C12	1.61609	1.35389	0.3549
C13	1.02246	1.07157	-0.13956
C14	1.14872	1.10079	-0.11799
N15	1.19167	1.07879	0.01778
C16	1.26185	1.12114	0.04873
C17	1.29367	1.19205	-0.06021
C18	1.36633	1.23446	-0.00981
C19	1.17789	1.17064	-0.23245
C20	1.25043	1.21745	-0.20341
C21	1.39416	1.03606	0.48906
O22	1.33738	0.98787	0.3942
O23	1.43757	1.02078	0.70096
C24	1.07237	1.04969	-0.1403
H25	1.27282	1.0399	0.28539
H26	1.52424	1.14257	0.55229
H27	1.57666	1.36932	0.33965
H28	1.03939	1.12682	-0.12622
H29	1.39343	1.28973	-0.07233
H30	1.14513	1.18866	-0.35568
H31	1.41589	0.96889	0.75597
H32	1.25786	1.37046	-0.40434

Section 4 Characterization of QL-COF-1&2

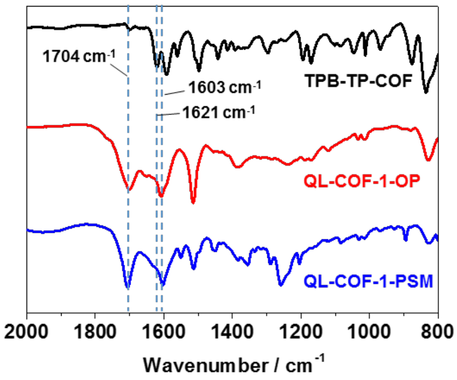

Supplementary Figure 2. Comparison of FT-IR spectra of TPB-TP-COF, QL-COF-1-OP and QL-COF-1-PSM.

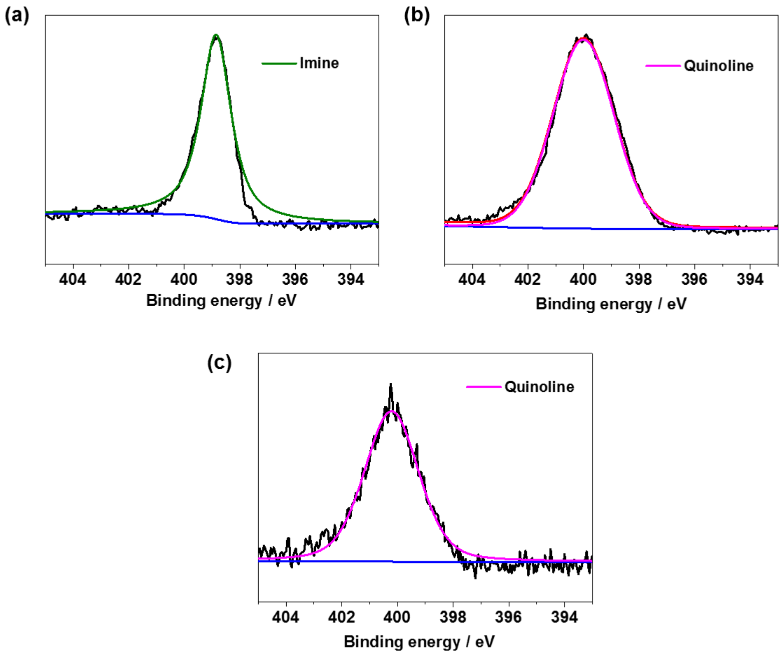

Supplementary Figure 3. XPS N 1*s* spectra of TPB-TP-COF (a), QL-COF-1-OP (b) and QL-COF-1-PSM (c).

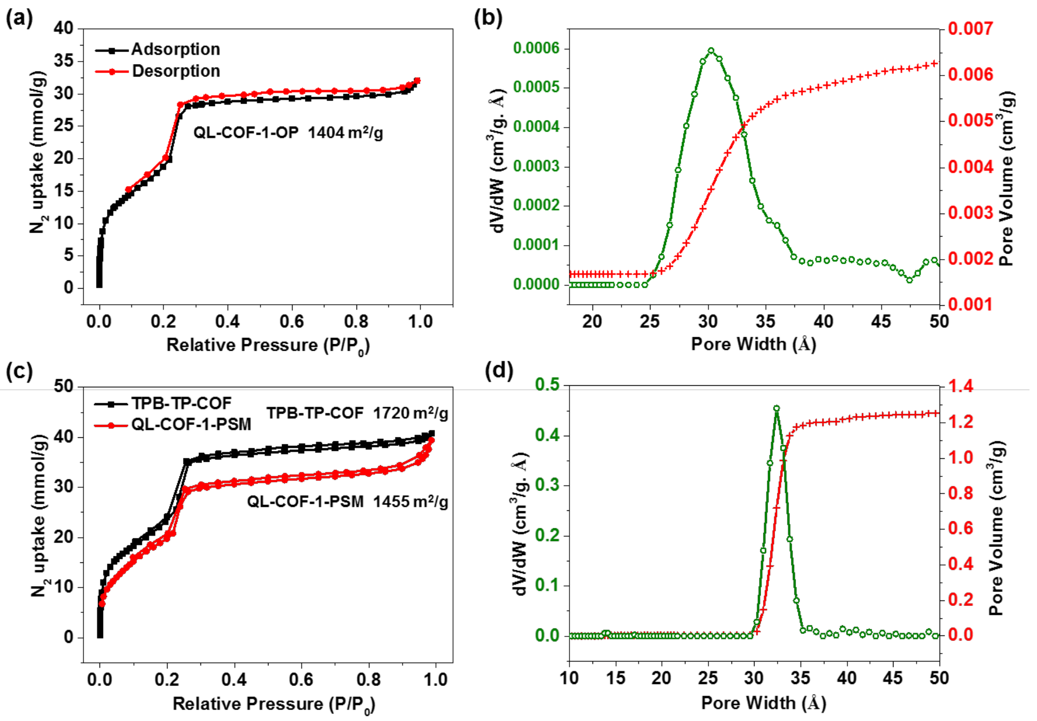

Supplementary Figure 4. Nitrogen adsorption experiment. N_2 adsorption isotherm of QL-COF-1-OP (a), TPB-TP-COF and QL-COF-2-PSM (c) and pore size distribution of QL-COF-1-OP (b), TPB-TP-COF (d) derived from NLDFT model.

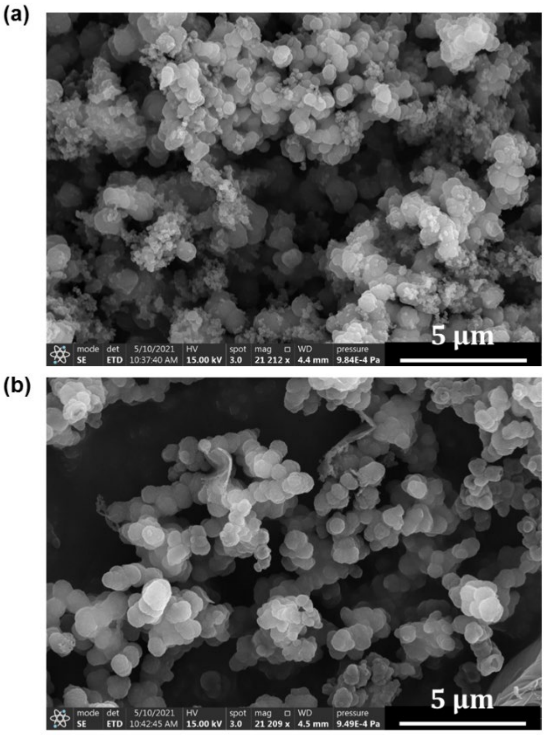

Supplementary Figure 5. SEM images of QL-COF-1-OP (a) and TPB-TP-COF (b).

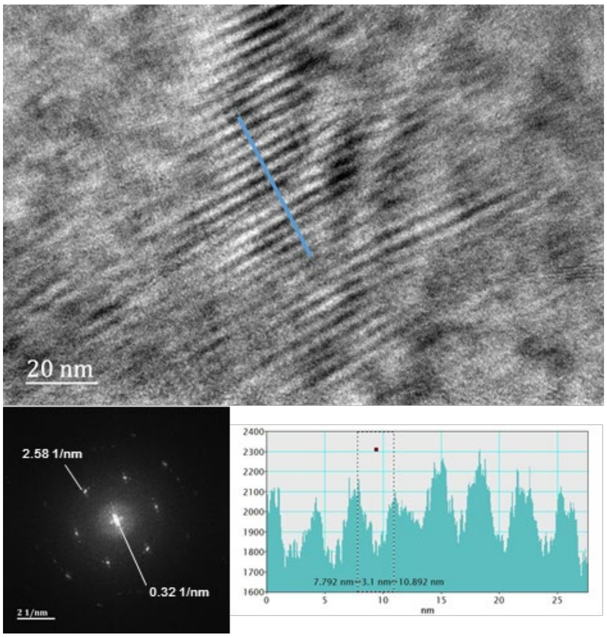

Supplementary Figure 6. HR-TEM image of QL-COF-1-OP. The interplanar d-spacing was ~3.0 nm.

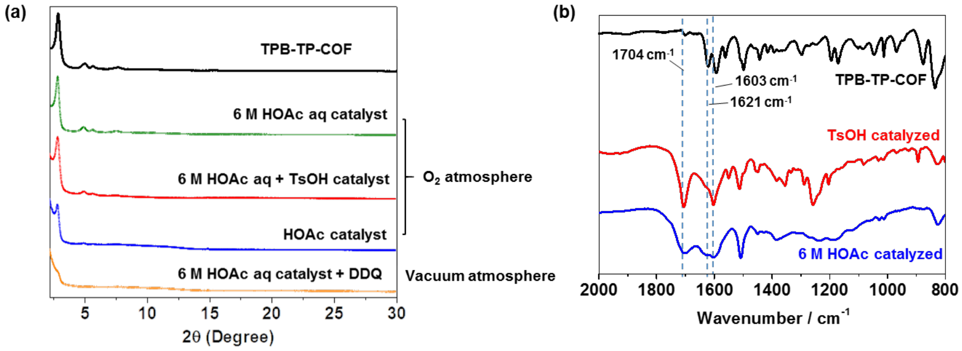

Supplementary Figure 7. Optimization of synthesis conditions for QL-COF-1-PSM. PXRD patterns(a) and IR spectra (b) of QL-COF-1-PSM product in different synthsis conditions.

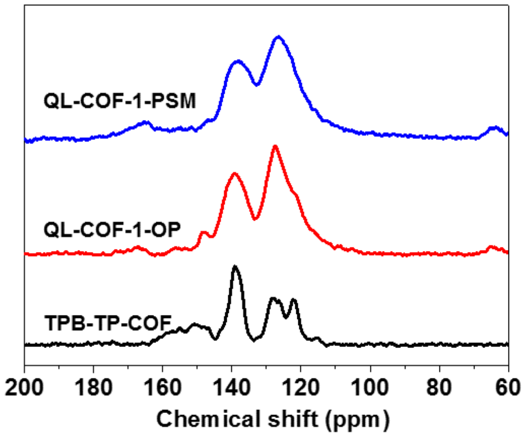

Supplementary Figure 8. ^{13}C NMR spectra of QL-COF-1-OP, QL-COF-1-PSM and TPB-TP-COF.

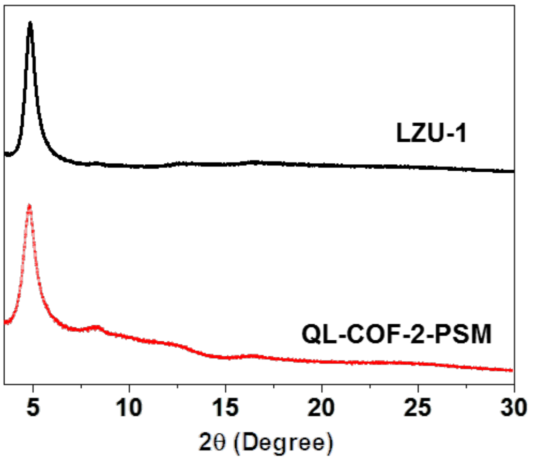

Supplementary Figure 9. Comparison of PXRD patterns of LZU-1 and QL-COF-2-PSM.

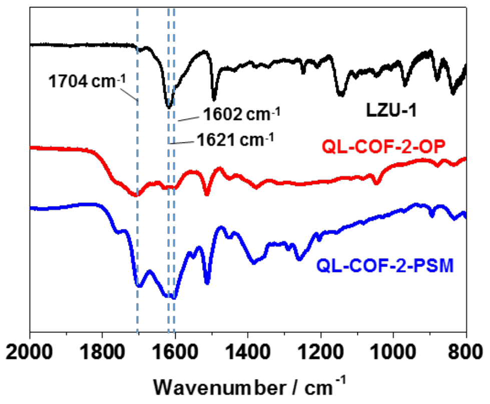

Supplementary Figure 10. Comparison of FT-IR spectra of LZU-1 (black), QL-

COF-2-OP and QL-COF-2-PSM.

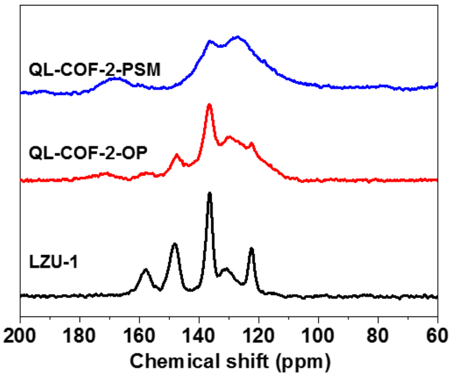

Supplementary Figure 11. Comparison of ^{13}C NMR spectra of LZU-1, QL-COF-2-OP and QL-COF-2-PSM.

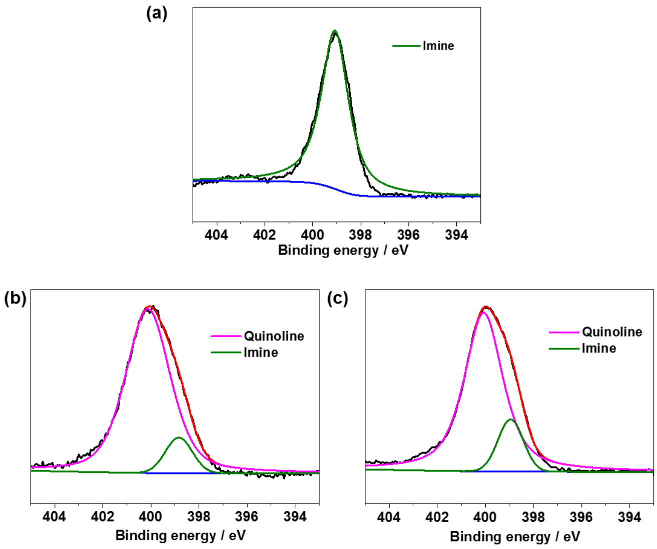

Supplementary Figure 12. XPS N 1*s* spectra of LZU-1 (a), QL-COF-2-OP/PSM (b, c).

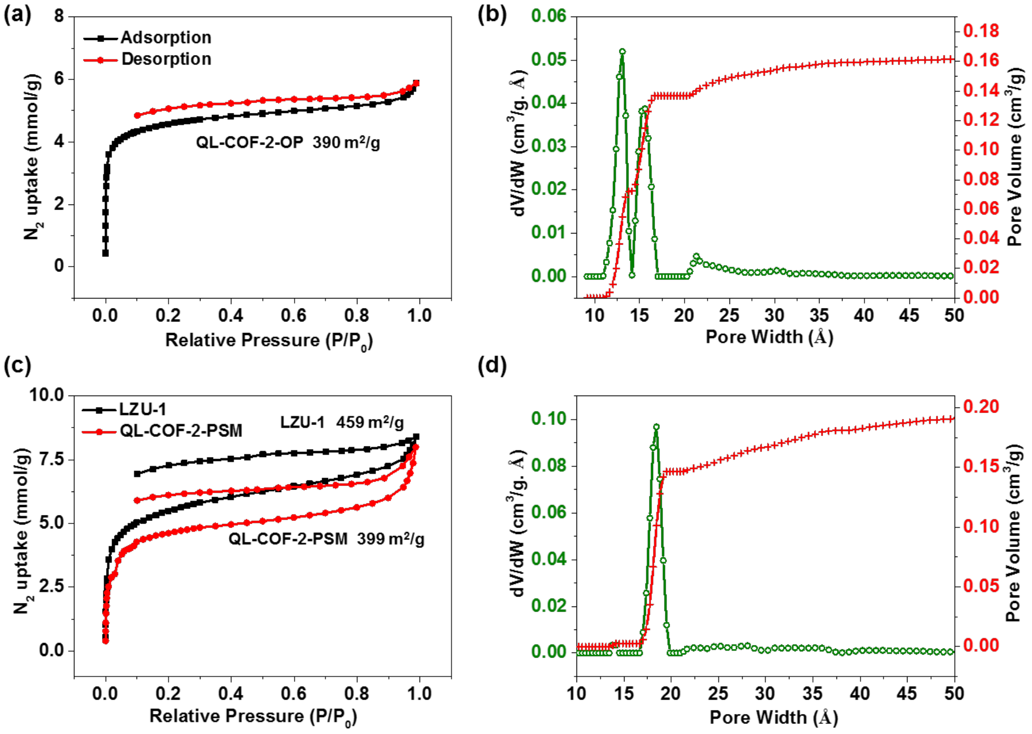

Supplementary Figure 13. Nitrogen adsorption experiment. N_2 adsorption isotherm of QL-COF-2-OP (a), LZU-1 and QL-COF-2-PSM (c) and pore size distribution of QL-COF-2-OP (b), LZU-1 (d) derived from NLDFT model.

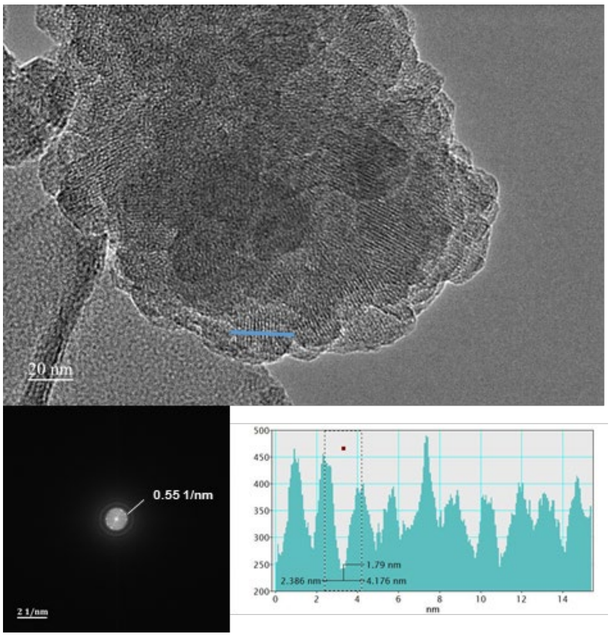

Supplementary Figure 14. HR-TEM image of QL-COF-2-OP. The interplanar d-spacing of ~0.36 nm was corresponding to the 001 lattice plane.

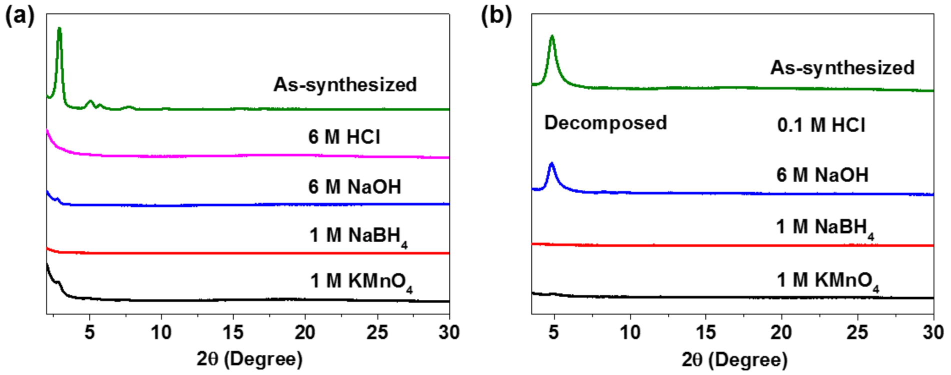

Supplementary Figure 15. PXRD patterns of TPB-TP-COF (a) and LZU-1-COF (b) after processing under relevant conditions.

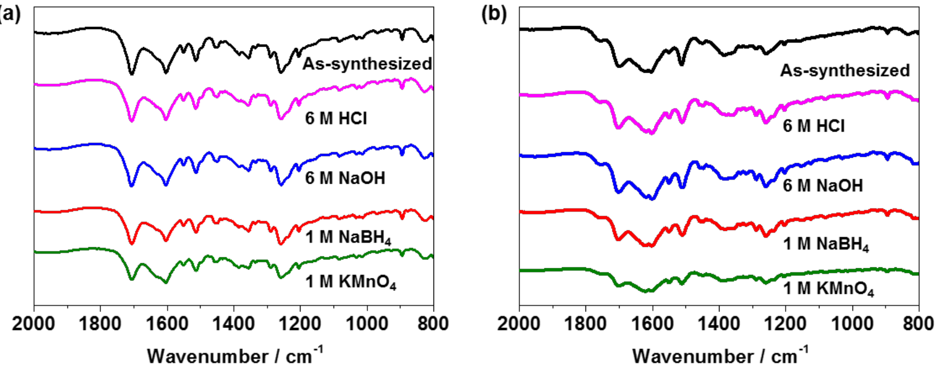

Supplementary Figure 16. FT-IR spectra of QL-COF-1 (a) and QL-COF-2 (b) after processing under relevant conditions.

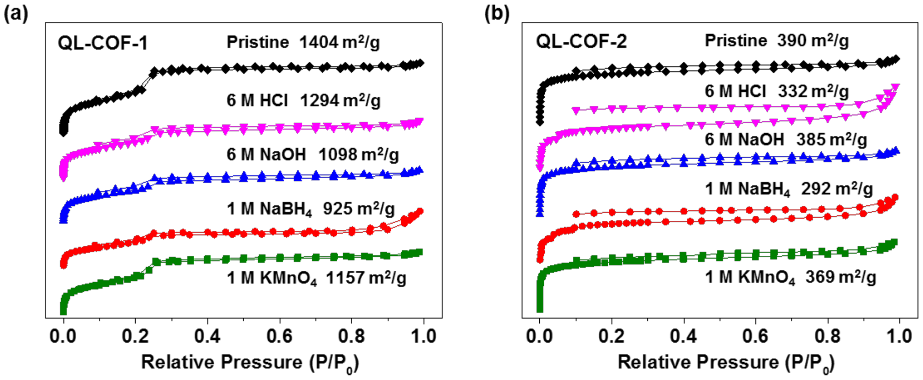

Supplementary Figure 17. Adsorption isotherm and BET surface area of QL-COF-1 (a) and QL-COF-2 (b) after processing under relevant conditions.

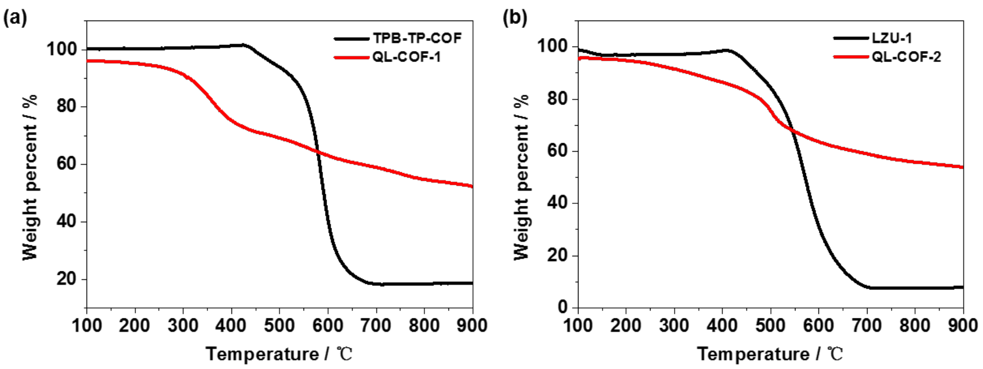

Supplementary Figure 18. (a) Comparision of TGA curves between TPB-TP-COF and QL-COF-1. (b) Comparision of TGA curves between LZU-1 and QL-COF-2.

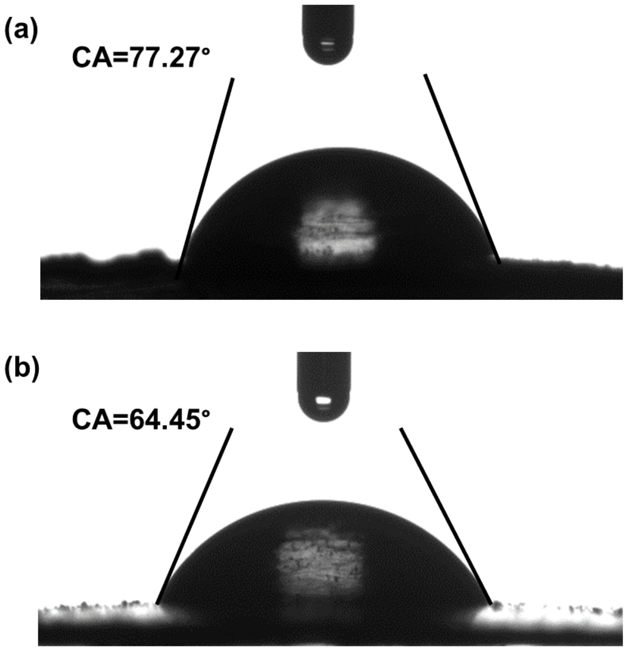

Supplementary Figure 19. Droplet contact angle measurements of LZU-1 (a) and QL-COF-2 (b).

Section 5 Characterization and nanofiltration of QL-COFs membranes

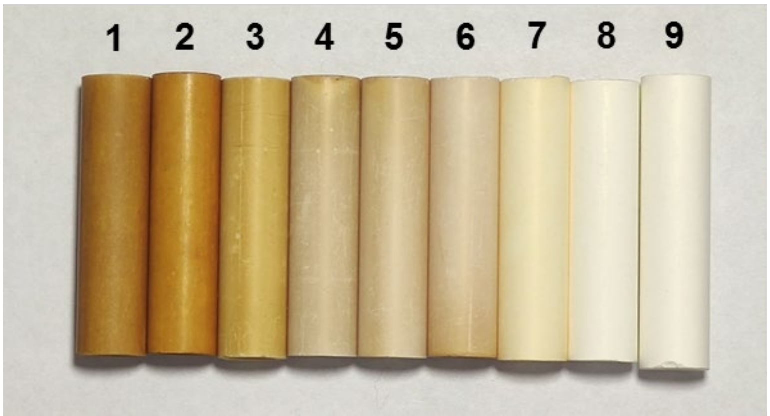

Supplementary Figure 20 Image of QL-COF-2 membranes@Al_2O_3 tube numbered as 1-7, corresponding to different concentration of the mother solution of 60, 20, 10, 6.25, 2.5, 1.25, 0.625 mmol/L, respectively. No. 8 is the APTES modified Al_2O_3 tube, No. 9 is the blank tube.

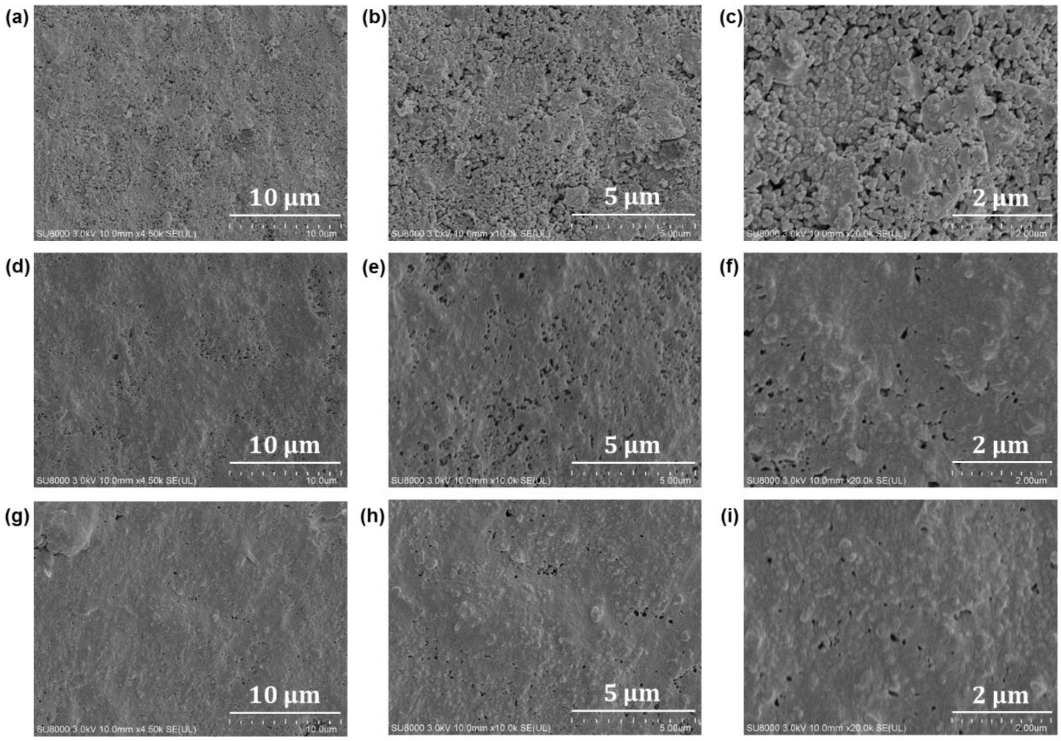

Supplementary Figure 21 SEM images of the Al_2O_3 membranes surface. a-c, pristine Al_2O_3 membranes, d-f, Al_2O_3 membranes after acidification, g-i, Al_2O_3 membranes after modified APTES.

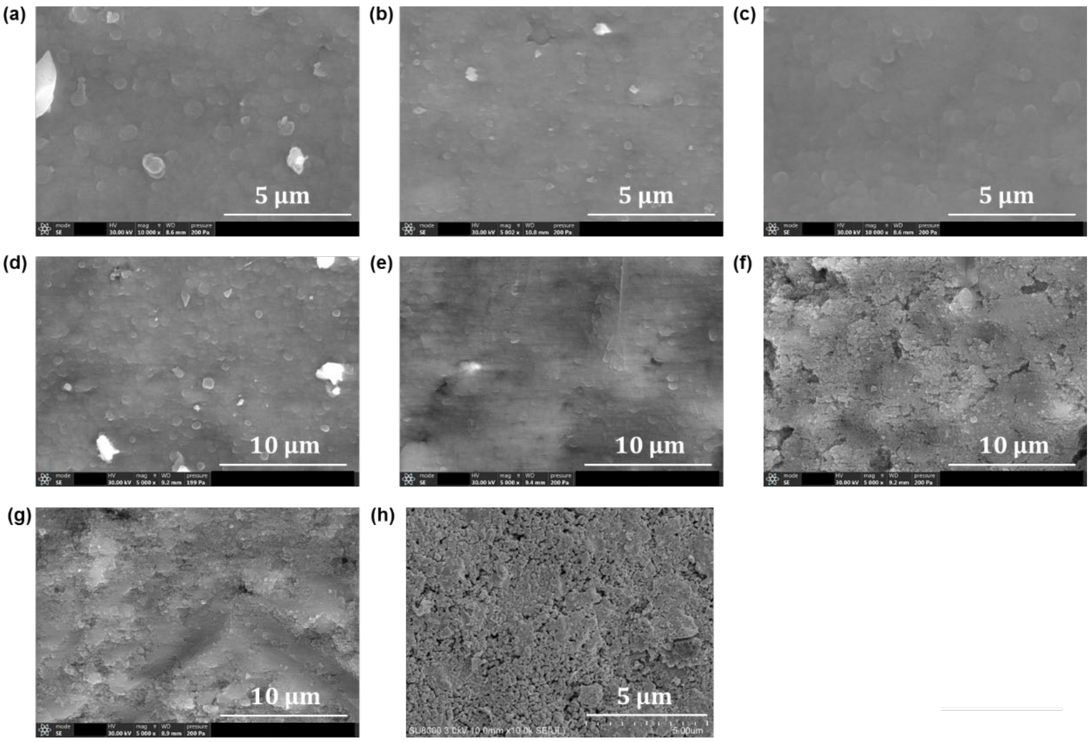

Supplementary Figure 22. SEM images of the QL-COF-2 membranes surface. Figure (a)-(g) corresponded to the membrane tubes of No. 1-7 in Supplementary Fig. 20. Figure (h) is the blank tube.

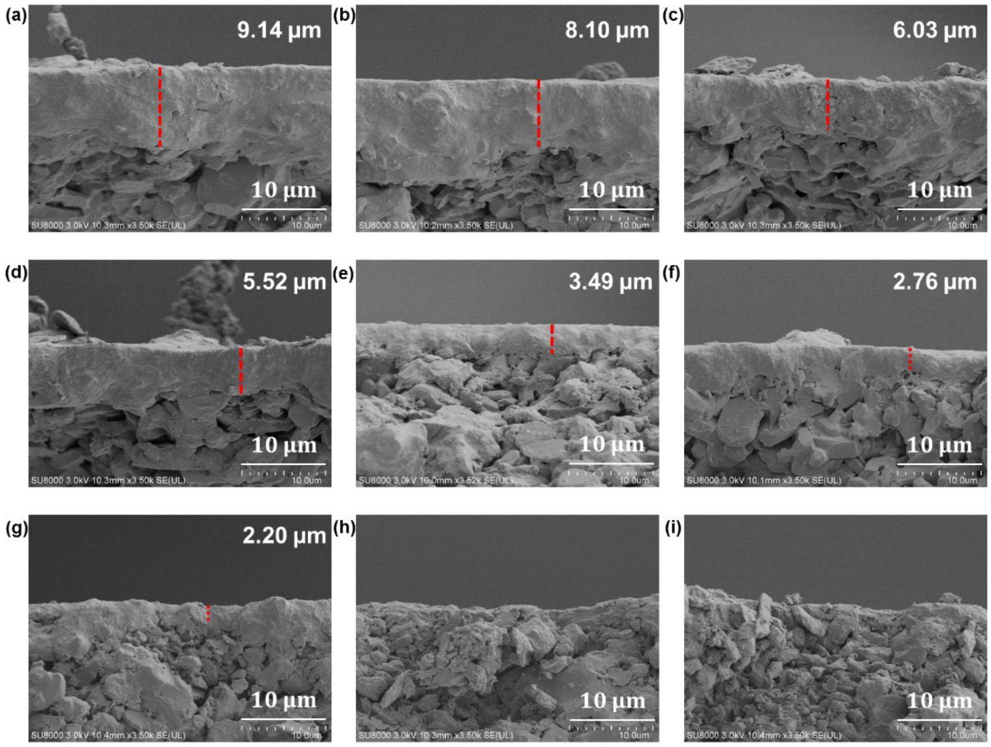

Supplementary Figure 23. The cross section SEM image of the membrane tubes. Figure (a)-(g) corresponded to the membrane tubes of No. 1-7 in Supplementary Fig.

20. Figure (h-i) are the Al_2O_3 membranes after acidification (h) and blank tube (i).

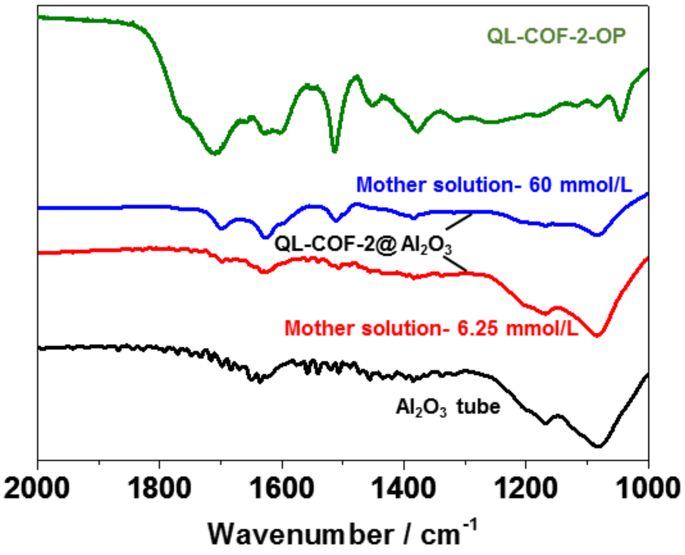

Supplementary Figure 24. IR spectra of the Al_2O_3 substrate, QL-COF-2 membrane synthesized with mother solution concentration of 60 and 6.25 mmol/L, and the QL-COF-2-OP.

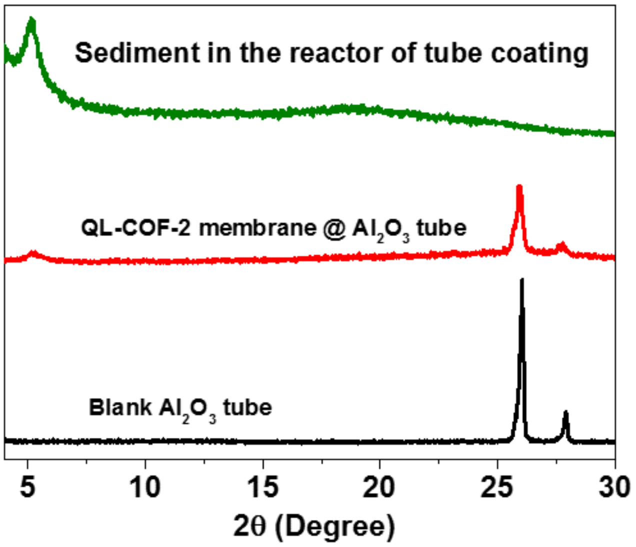

Supplementary Figure 25. PXRD patterns of the Al_2O_3 substrate, as-synthesized QL-COF-2 membrane and the sediment in the reactor of tube coating.

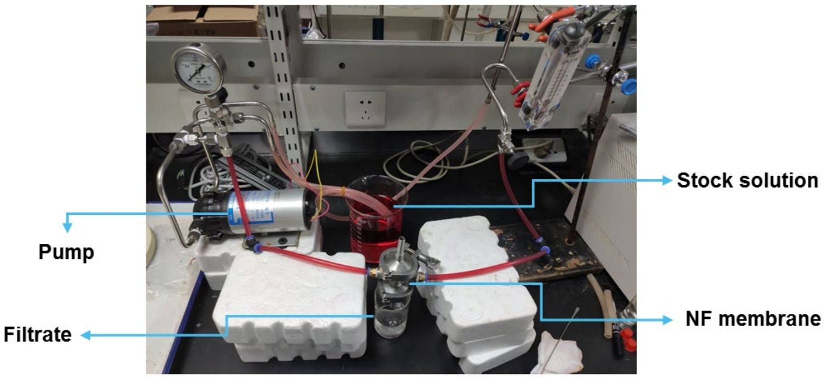

Supplementary Figure 26. The home-made cross-flow filtration NF equipment.

Determination of Congo red adsorption capacity by QL-COF-2

In a typical adsorption experiment, 5 mg of QL-COF-2 was dispersed with 15 mL of Congo red solution (50 mg L^{-1}). All samples were then capped and placed on an orbital shaker at 300 rpm for either 1, 2, 5, 10, 24, 48, or 72 h. After incubating for the specified amount of time, the samples were each removed from the shaker and centrifuged to remove COF powder. The resulting dye solutions were analyzed by UV-Vis spectroscopy at the specified wavelengths (λ_{max} = 504 nm) to determine the quantity of dye that had adsorbed to the COF powder at each time point.

The adsorption capacity of CR on QL-COF-2 was calculated by equation:

$$q_t = \frac{(c_0 - c_t) \times V}{m}$$

where q_t (mg g^{-1}) is the adsorption capacity of CR at time t, c_0 and c_t (mg L^{-1}) are the concentrations of CR at initial and time t (h). V (L) is the volume of CR solution, and m (g) is the dosage of QL-COF-2 powder.

The pseudo second-order kinetic model was further used to evaluate the adsorption of CR on QL-COF-2.

$$\frac{t}{q_t} = \frac{1}{kq_e^2} + \frac{t}{q_e}$$

where q_e and q_t are the adsorption capacity (mg g^{-1}) at equilibrium and time t (h). k is the pseudo-second-order rate constant (g mg^{-1} h^{-1}). The pseudo-second-order kinetics plots show good linearity for all the initial concentrations, suggesting the well-fitting of pseudo-second-order kinetic model for CR on QL-COF-2. **The q_e value was calculated as 40.88 g mg^{-1}** in the 50 mg L^{-1} CR solution.

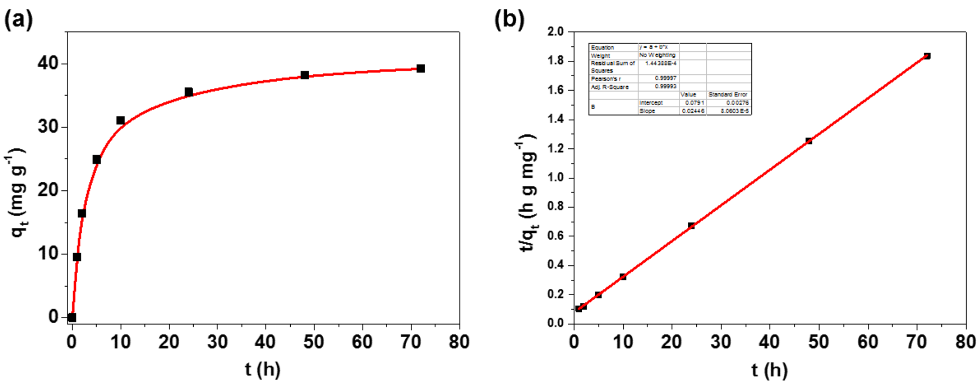

Supplementary Figure 27. Time-dependent adsorption capacity (a) and pseudo-second-order kinetics plots (b) for the adsorption of CR on QL-COF-2.

Single-point Dye Uptake Experiments

To probe the affinity of 10 kinds of dye molecules (1-neutral red, 2-methylene blue, 3 -methyl orange, 4-acid blue, 5-chrome black T, 6-acid fuchsin, 7-Congo red, 8-methyl blue, 9-rose bengal, 10-vitamin B-12) for QL-COF-2, the following experiments were performed. To each of ten 50 mL centrifuge tubes were added QL-COF-2 powder (5 mg). Next, each vial was filled with 10 kinds of dye solutions (50 mg/L, 50mL), respectively. All samples were then capped and placed on an orbital shaker at 300 rpm for 72 hours to ensure that equilibrium was reached. Next, the COFs powder was separated by centrifugation. The resulting dye solutions were then diluted tenfold and analyzed by UV-Vis spectroscopy to determine the removal percent of each dye that had adsorbed by the QL-COF-2.

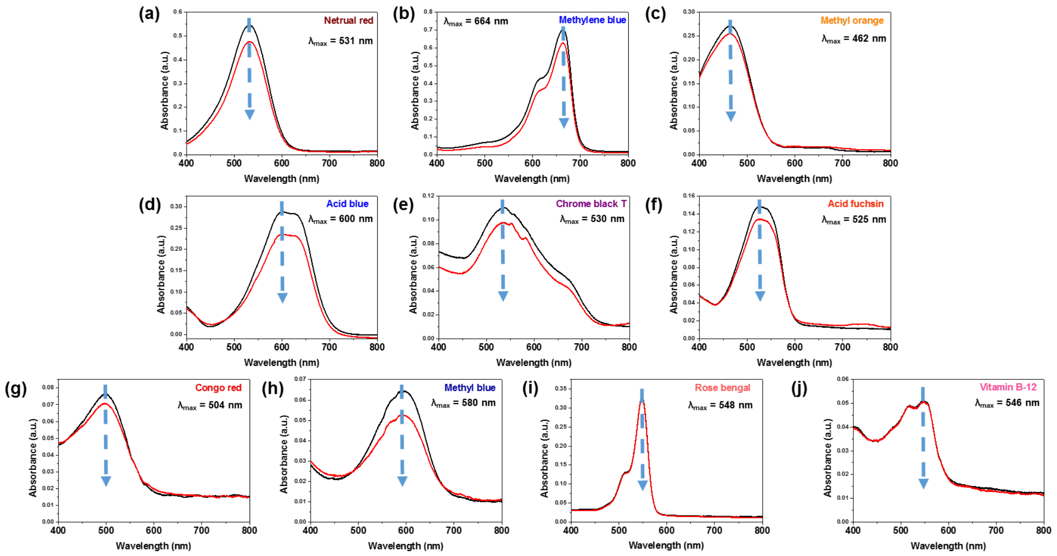

Supplementary Figure 28. UV-Vis spectra of (a) netrual red, (b) methylene blue, (c) methyl orange, (d) acid blue, (e) chrome black T, (f) acid fuchsin, (g) Congo red, (h) methyl blue, (i) rose bengal, (j) vitamin B-12 dyes solution before and after the single-point dye uptake experiments.

Supplementary Table 5. Single Point Uptake Data for 10 kinds of dyes. (50 mg L^{-1} dye solution, 100 mg L^{-1} COF loading)

Serial number of dye	λ_{max} (nm)	Absorbance before Adsorption experiments	Absorbance after Adsorption experiments	Removal %
1	531	0.54667	0.47623	12.89
2	664	0.70599	0.62838	10.99
3	462	0.27067	0.25503	5.78
4	600	0.2879	0.23549	18.2
5	530	0.10969	0.09722	11.37
6	525	0.14826	0.13443	9.33
7	504	0.07589	0.06994	7.84
8	580	0.06219	0.05067	18.52
9	548	0.33009	0.32822	0.57
10	546	0.05115	0.05067	0.94

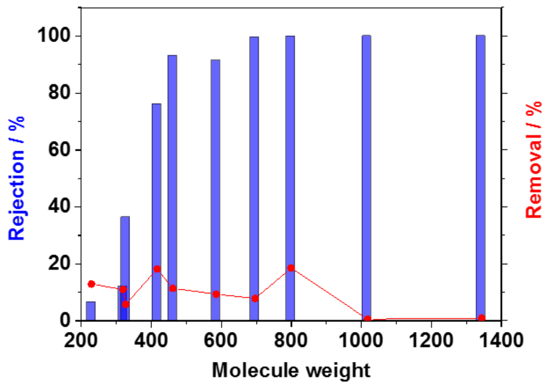

Supplementary Figure 29. The adsorption affinity (red) and rejection rate (blue) in nanofiltration of QL-COF-2 for ten dyes with different molecular weights.

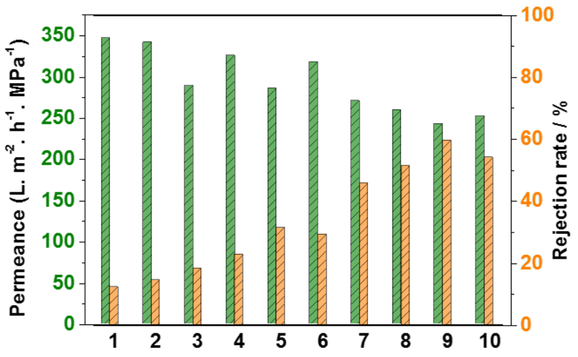

Supplementary Figure 30. Rejection rates of various dyes and the water permeances of QL-COF-1 membrane.

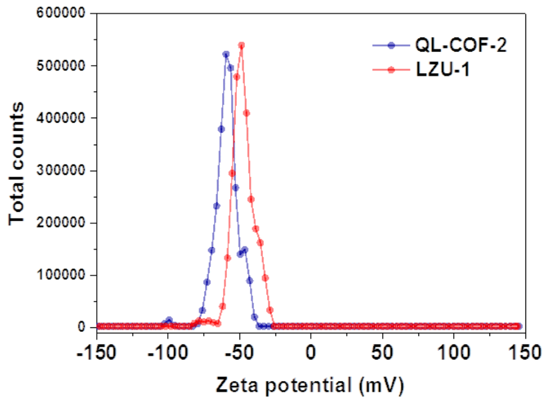

Supplementary Figure 31. Zeta potential distribution of QL-COF-2 under pH=7.

Section 6 Ion Sieving experiments of QL-COF-2 membrane

Membrane fabrication for cation separation

QL-COF-2 powder was sufficiently exfoliated in basic water (0.1 M NaOH) by sonication in cell crusher for 12 h. Sequentially, the bulk COF was separated by centrifugation in 4000 rpm and the supernatant was collected. The COF dispersion liquid was then poured onto the AAO membrane (Whatman, pore size 0.2 μm) to form QL-COF-2 membrane via vacuum filtration under 0.6 bar pressure. The as-prepared QL-COF-2/AAO membranes were washed by 1 M HCl, water and methanol and drying under vacuum at 60 °C for 12 h. The difference value between the mass of the composite QL-COF-2/AAO membranes and the blank AAO membrane were the mass of the COF membrane. In this work, three QL-COF-2/AAO membranes were prepared with different mass (~ 2, 10, 20 mg) of QL-COFs, denoting as QL-COF-2 (2)/(10)/(20).

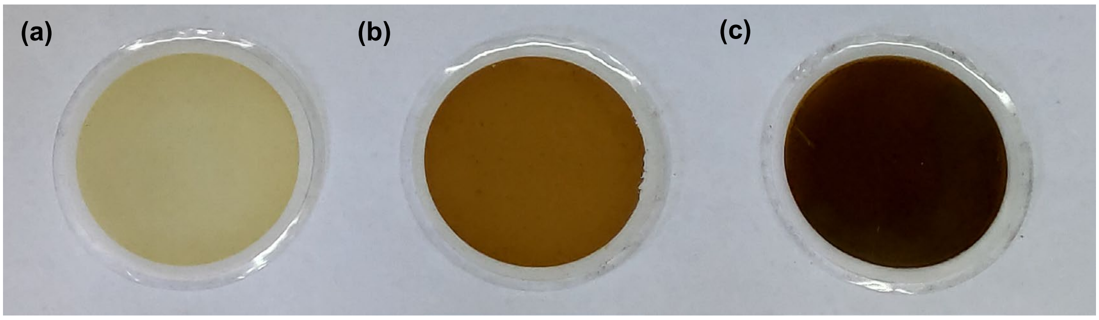

Supplementary Figure 32. Photograph of QL-COF-2/AAO membrane. (a) QL-COF-2 (2), (b) QL-COF-2 (10), (c) QL-COF-2 (20).

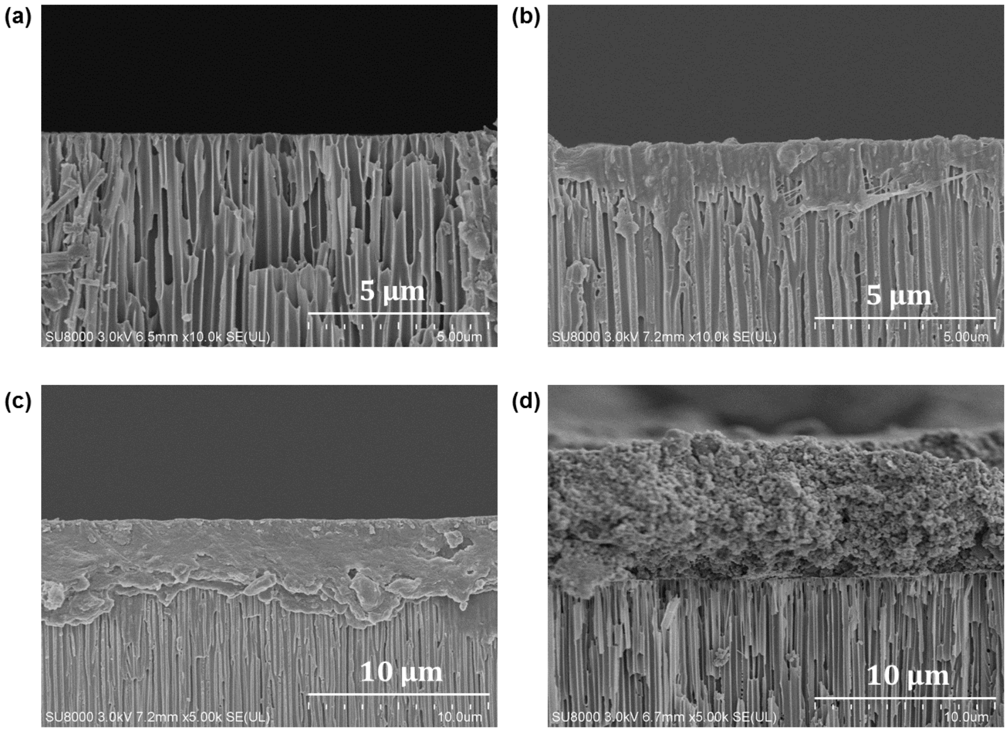

Supplementary Figure 33. The cross section SEM images of the membranes. (a) Blank AAO membrane (0.2 µm pore size), (b) QL-COF-2 (2), (c) QL-COF-2 (8), (d) QL-COF-2 (20).

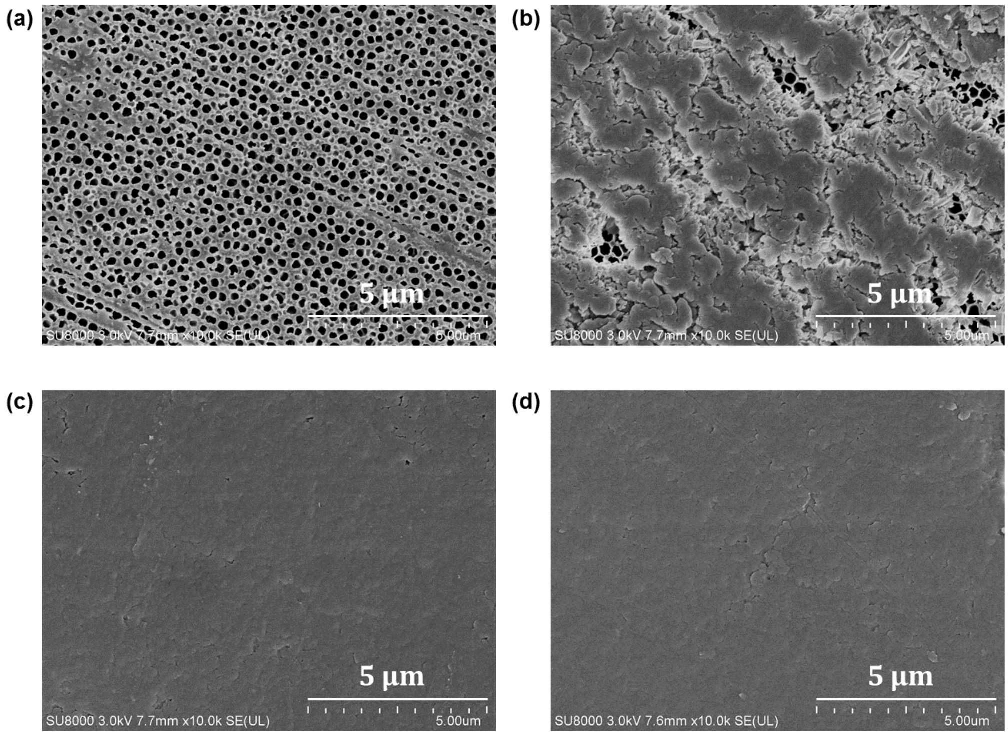

Supplementary Figure 34. SEM images of the membranes surface. (a) Blank AAO membrane (0.2 µm pore size), (b) QL-COF-2 (2), (c) QL-COF-2 (10), (d) QL-COF-2

(20).

Cationic selective transmembrane experiments

The cation selectivity of QL-COF-2 membrane was investigated by measuring their conductivity of various cations between the both sides of the membrane. Various tetraalkyl ammonium polystyrenesulfonate salts with different sizes of cations, including tetramethylammonium (Me_4N^+), tetraethylammonium (Et_4N^+), tetrabutylammonium (Bu_4N^+), tetrahexylammonium (Hex_4N^+), tetraoctylammonium (Oct_4N^+) and tetradodecylammonium ($Dodec_4N^+$), were synthesized according to the literature.[4]

The apparatu for ion selectivity measurement was a H-shaped tube divided by a membrane into two compartments (Supplementary Fig. 35). Both sides were filled with 0.001M of different ammonium solutes in methanol (OPTIMA LC-MS, Fisher Chemical) with 50 mL volume. A platinum sheet electrode (0.5*0.5 cm) was inserted in each compartment, which is connected to a electrochemical workstation (CHI 760E, Shanghai Chenhua). Ion transport can be measured by recording I-V current for a voltage range of 0-2 V. The slope of the linear I−V curves was used to define the transmembrane selectivity of the cations.

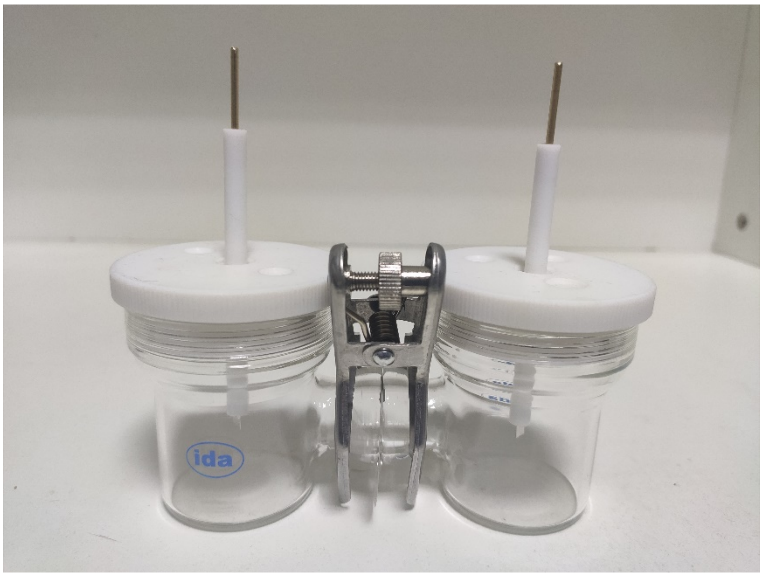

Supplementary Figure 35. Conductivity apparatus for ion selectivity measurements

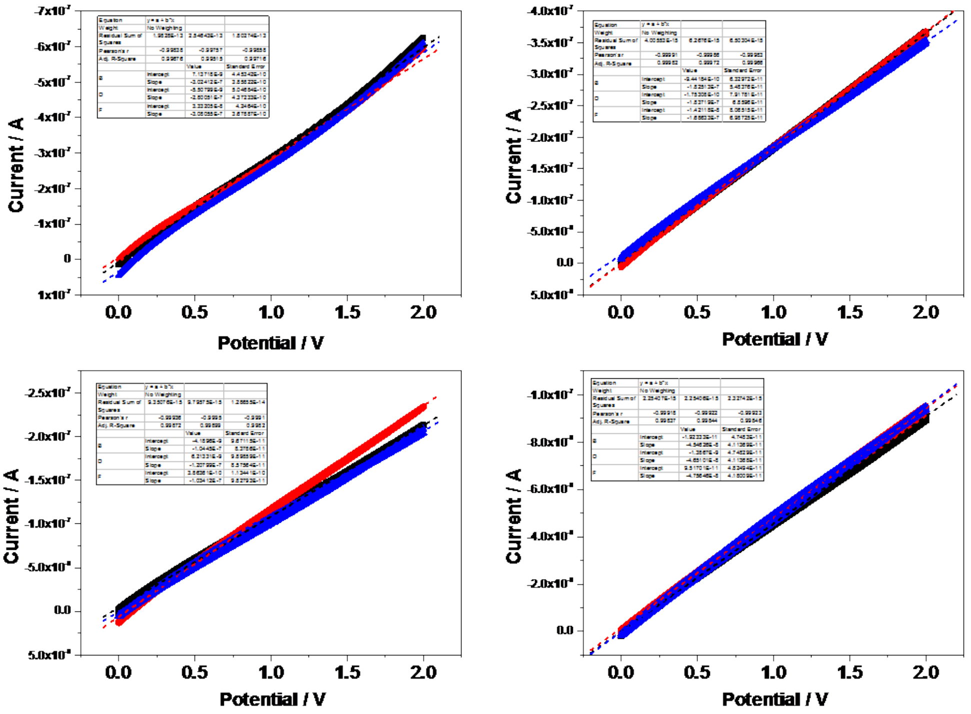

Supplementary Figure 36. I-V curves of tetramethylammonium (Me_4N^+) transport of AAO membrane (a) and QL-COF-2 (10, 20, 50) (b-d).

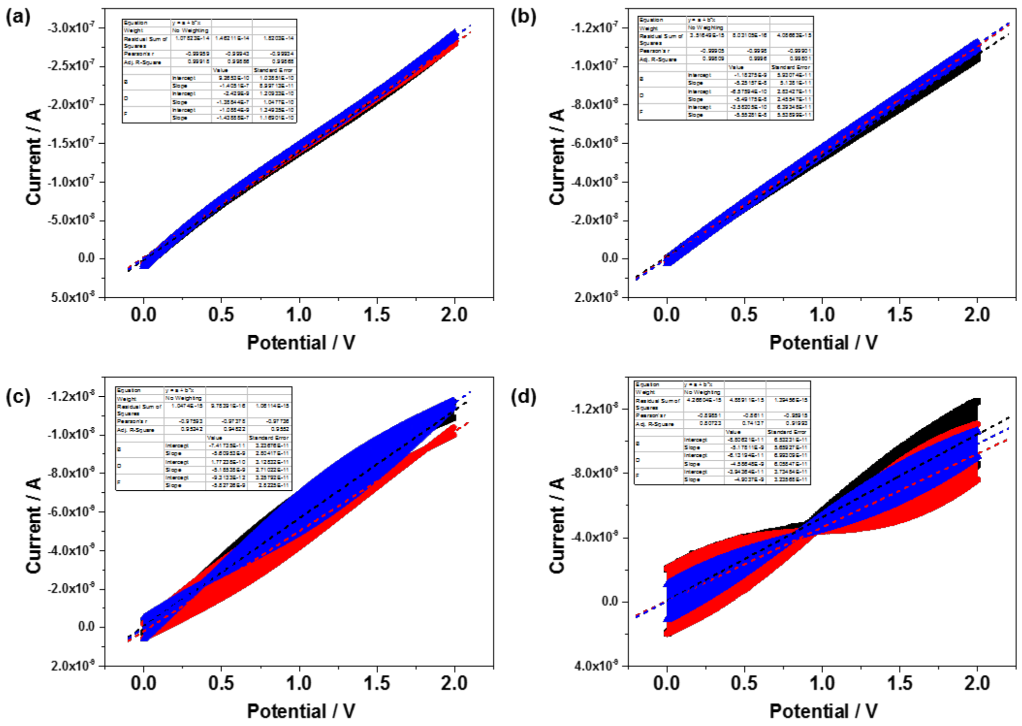

Supplementary Figure 37. I-V curves of tetradodecylammonium ($Dodec_4N^+$) transport of AAO membrane (a) and QL-COF-2 (10, 20, 50) (b-d).

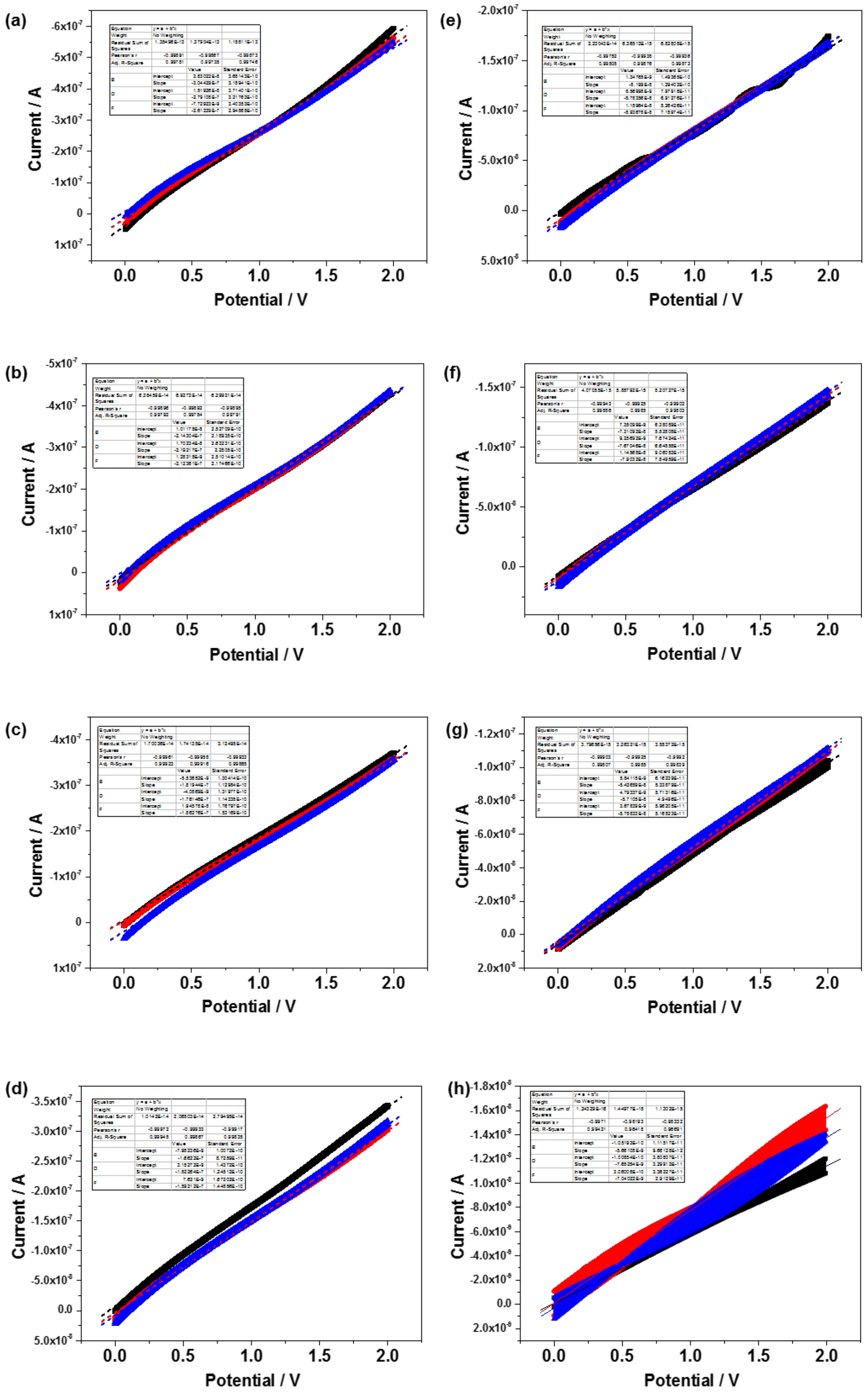

Supplementary Figure 38. I-V curves of alkyl ammonium salts transport of AAO membrane (a-d) and QL-COF-2 (20) membrane (e-h). Figure a, e-tetraethylammonium

(Et_4N^+); b, f-tetrabutylammonium (Bu_4N^+); c, g-tetrahexylammonium (Hex_4N^+); d, h-tetraoctylammonium (Oct_4N^+).

Supplementary Table 6. Conductivities of tetramethylammonium (Me_4N^+) and tetradodecylammonium ($Dodec_4N^+$) transport of membranes.

Cations	Membrane	G (μS)	Conductivity (μS cm^{-1})	avg (μS cm^{-1})
Me_4N^+	AAO	0.3024	8.58816	8.431
		0.2801	7.95484	
		0.3081	8.75004	
	QL-COF-2 (10)	0.1825	5.183	5.063
		0.1837	5.21708	
		0.1686	4.78824	
	QL-COF-2 (20)	0.1045	2.9678	3.112
		0.1208	3.43072	
		0.1034	2.93656	
	QL-COF-2 (50)	0.04546	1.291064	1.321
		0.04651	1.320884	
		0.04756	1.350704	
$Dodec_4N^+$	AAO	0.1405	3.9902	4.006
		0.1388	3.94192	
		0.1439	4.08676	
	QL-COF-2 (10)	0.05252	1.491568	1.543
		0.05492	1.559728	
		0.05553	1.577052	
	QL-COF-2 (20)	0.00561	0.159324	0.1574
		0.005185	0.147254	
		0.005827	0.1654868	
	QL-COF-2 (50)	0.005178	0.1470552	0.1389
		0.004586	0.1302424	
		0.004904	0.1392736	

Supplementary Table 7. Conductivities of alkyl ammonium salts (Et_4N^+, Bu_4N^+, Hex_4N^+ and Oct_4N^+) transport of membranes.

Cation	Membrane	G (μS)	Conductivity (μS cm^{-1})	avg (μS cm^{-1})
Et_4N^+	AAO	0.3044	8.645	7.996
		0.2791	7.926	
		0.2612	7.418	
	QL-COF-2 (20)	0.08199	2.329	2.454
		0.08784	2.495	
		0.08937	2.538	
Bu_4N^+	AAO	0.2143	6.086	6.115
		0.2192	6.225	
		0.2124	6.032	
	QL-COF-2 (20)	0.07311	2.076	2.166
		0.0767	2.178	
		0.07903	2.244	
Hex_4N^+	AAO	0.1819	5.166	5.153
		0.1761	5.001	
		0.1863	5.291	
	QL-COF-2 (20)	0.05427	1.541	1.599
		0.05711	1.622	
		0.05758	1.635	
Oct_4N^+	AAO	0.1662	4.720	4.522
		0.1523	4.325	
		0.1592	4.521	
	QL-COF-2 (20)	0.005661	0.1608	0.1927
		0.007653	0.2173	
		0.00704	0.1999	

References

1. Waller PJ, Lyle SJ, Osborn Popp TM, Diercks CS, Reimer JA, Yaghi OM. Chemical Conversion of Linkages in Covalent Organic Frameworks. *J Am Chem Soc* **138**, 15519-15522 (2016).

2. Ding S-Y*, et al.* Construction of Covalent Organic Framework for Catalysis: Pd/COF-LZU1 in Suzuki-Miyaura Coupling Reaction. *J Am Chem Soc* **133**, 19816-19822 (2011).

3. Wang X*, et al.* Design, synthesis and antibacterial evaluation of some new 2-phenyl-quinoline-4-carboxylic acid derivatives. *Molecules* **21**, 340 (2016).

4. Bhiwankar NN, Weiss R. Melt intercalation/exfoliation of polystyrene–sodium-montmorillonite nanocomposites using sulfonated polystyrene ionomer compatibilizers. *Polymer* **47**, 6684-6691 (2006).
